# Analysis of Immune–Stromal Score-Based Gene Signature and Molecular Subtypes in Osteosarcoma: Implications for Prognosis and Tumor Immune Microenvironment

**DOI:** 10.3389/fgene.2021.699385

**Published:** 2021-09-23

**Authors:** Dingzhao Zheng, Kaichun Yang, Xinjiang Chen, Yongwu Li, Yongchun Chen

**Affiliations:** ^1^ Department of Rehabilitation Medicine, The Fifth Hospital of Xiamen, Xiamen, China; ^2^ Emergency Department, The Fifth Hospital of Xiamen, Xiamen, China; ^3^ Department of Orthopaedics, The Fifth Hospital of Xiamen, Xiamen, China

**Keywords:** osteosarcoma, immune score, stromal score, gene signature, prognosis, molecular subtype

## Abstract

**Objective:** Infiltrating immune and stromal cells are essential for osteosarcoma progression. This study set out to analyze immune–stromal score-based gene signature and molecular subtypes in osteosarcoma.

**Methods:** The immune and stromal scores of osteosarcoma specimens from the TARGET cohort were determined by the ESTIMATE algorithm. Then, immune-stromal score-based differentially expressed genes (DEGs) were screened, followed by univariate Cox regression analysis. A LASSO regression analysis was applied for establishing a prognostic model. The predictive efficacy was verified in the GSE21257 dataset. Associations between the risk scores and chemotherapy drug sensitivity, immune/stromal scores, PD-1/PD-L1 expression, immune cell infiltrations were assessed in the TARGET cohort. NMF clustering analysis was employed for characterizing distinct molecular subtypes based on immune-stromal score-based DEGs.

**Results:** High immune/stromal scores exhibited the prolonged survival duration of osteosarcoma patients. Based on 85 prognosis-related stromal–immune score-based DEGs, a nine-gene signature was established. High-risk scores indicated undesirable prognosis of osteosarcoma patients. The AUCs of overall survival were 0.881 and 0.849 in the TARGET cohort and GSE21257 dataset, confirming the well predictive performance of this signature. High-risk patients were more sensitive to doxorubicin and low-risk patients exhibited higher immune/stromal scores, PD-L1 expression, and immune cell infiltrations. Three molecular subtypes were characterized, with distinct clinical outcomes and tumor immune microenvironment.

**Conclusion:** This study developed a robust prognostic gene signature as a risk stratification tool and characterized three distinct molecular subtypes for osteosarcoma patients based on immune–stromal score-based DEGs, which may assist decision-making concerning individualized therapy and follow-up project.

## Introduction

Osteosarcoma represents the most frequent and primary bone sarcoma, which primarily affects children, adolescents, and young adults (Corre et al., 2020). Globally, the incidence of osteosarcoma is approximately 1–3 cases yearly per million persons (Itoh et al., 2018). Neo-adjuvant therapy followed by postoperative adjuvant therapy with a cocktail of chemotherapy is the first-line therapeutic strategy for locally resectable osteosarcoma, leading to increased 5-years survival rate that is up to 70% for patients with localized osteosarcoma (Deng et al., 2020). However, most of metastatic or relapsed patients do not benefit from this therapy and the 5-years survival rate is below 30% (Yang et al., 2020). How to improve osteosarcoma treatment and prolong clinical outcomes is a prime issue in current research.

Osteosarcoma is characterized by huge heterogeneity at the intra-tumoral and individual levels (Zhou et al., 2020b). Thus, it is of significance to identify shared gene sets driving osteosarcoma. Tumor microenvironment is composed of mesenchymal cells, immune cells, endothelial cells, stromal cells, inflammatory mediators, and the like (Zhang et al., 2020). It participates in mediating biological properties of osteosarcoma like metastasis, immune escape, and drug resistance (Zheng et al., 2018). Evidence demonstrates that immune- and stroma cell-related signatures in the tumor microenvironment serve as critical regulators in the prognosis of osteosarcoma patients (Wen et al., 2020). Precise management and proper individualized therapy regarding osteosarcoma are required in conformity to risk stratification. Furthermore, it is of importance to understand the immune-stromal score-based signatures in the tumor immune microenvironment, which may assist clarify clinical implications in the tumor immune microenvironment as well as propose novel therapeutic strategies (Qi et al., 2020). Herein, this study set out to characterize immune–stromal score-based gene signature as a prognostic stratification tool and molecular subtypes with distinct prognosis and tumor immune microenvironment in osteosarcoma.

## Materials and Methods

### Data Preparation and Preprocessing

**FIGURE 1:**
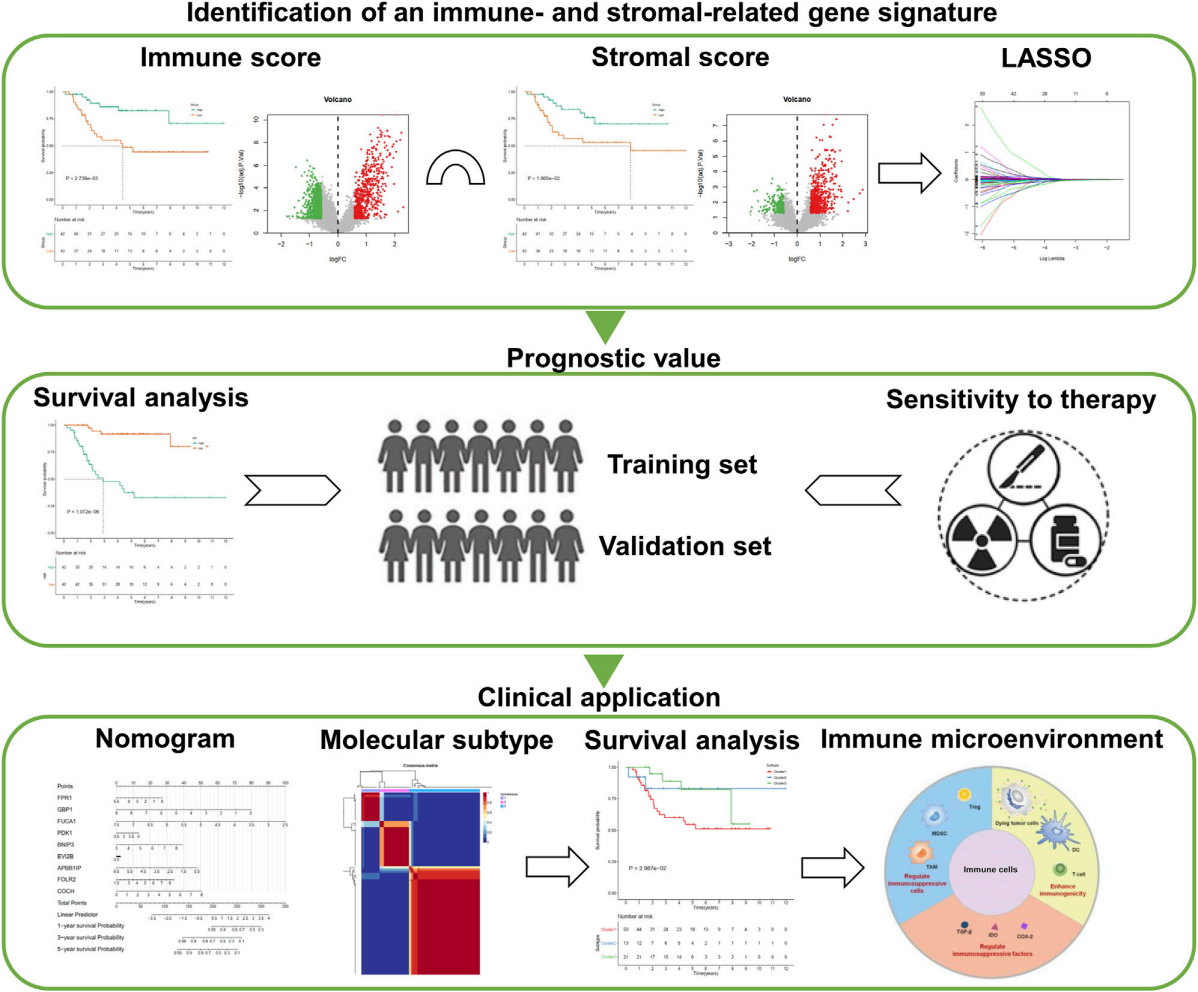
Study design and workflow overview.

Clinical and transcriptome data of osteosarcoma patients were downloaded from the Therapeutically Applicable Research to Generate Effective Treatments (TARGET) database (https://ocg.cancer.gov/programs/target). Eighty-four samples with both complete survival information and expression profiles were set as the training set. One external dataset from the Gene Expression Omnibus (GEO; https://www.ncbi.nlm.nih.gov/geo/) was selected as the validation set (accession: GSE21257). This dataset contained 53 osteosarcoma samples on the GPL10295 platform. The specific clinical information of 84 samples in the TARGET database and 53 samples in the GSE21257 dataset was separately listed in Supplementary Tables S1 and S2. The workflow of this study is shown in Figure 1.

### Estimation of STromal and Immune Cells in MAlignant Tumor Tissues Using Expression Data

Based on the normalized expression matrix, stromal and immune scores across osteosarcoma specimens from the TARGET and GSE21257 datasets were estimated by applying the ESTIMATE algorithm (Yoshihara et al., 2013). This algorithm may infer the overall infiltration levels of stromal and immune cells in tumor tissues using gene expression signatures (https://sourceforge.net/projects/estimateproject/). On the basis of the median values of stromal/immune scores, the patients were separated into two groups, respectively. The Kaplan–Meier overall survival (OS) curves were examined between groups and the prognosis was compared by log-rank test.

### Differential Expression Analysis

The limma package was applied for differential expression analysis between high and low stromal/immune score groups of osteosarcoma samples from the TARGET database (Ritchie et al., 2015). |Fold change (FC)| > 1.5 and adjusted *p* < 0.05 were set as the criteria of differentially expressed gene (DEG) identification. The up or downregulated genes were visualized into volcano plots. Upregulated genes from immune/stromal high versus low groups and downregulated genes were separately intersected.

### Functional Enrichment Analysis

The enrichment analysis of stromal-immune score-based DEGs was carried out via the clusterProfiler package, including Gene Ontology (GO) and Kyoto Encyclopedia of Genes and Genomes (KEGG) (Yu et al., 2012). GO categories contained biological process (BP), molecular function (MF), as well as cellular component (CC). Terms with adjusted *p* < 0.05 were significantly enriched.

### Construction of a Stromal-Immune Score-based Gene Signature

Correlations between the 272 stromal-immune score-based DEGs and prognosis of osteosarcoma patients from the TARGET database were evaluated by univariate Cox regression analysis. *p* < 0.05 was set as the significant cut-off for identifying candidate genes related to osteosarcoma prognosis. The least absolute shrinkage and selection operator (LASSO) was employed for feature selection and obtaining an optimal gene signature in the TARGET database. By applying 10-fold cross-verification and penalty, a prognostic gene signature was constructed *via* the glmnet package. The risk score of each sample from the training and validation sets was determined based on expression profiles and coefficients of feature genes. The formula was as follows: risk score = expression of gene 1 × coefficient of gene 1 + expression of gene 2 × coefficient of gene 2 +…+ expression of gene n × coefficient of gene n.

### Evaluation of the Prognostic Efficacy of the Gene Signature

The osteosarcoma patients in the training and validation sets were classified into high- and low-risk groups on the basis of the median value of the risk scores. The OS time between groups was compared by Kaplan–Meier curves and log-rank test. Receiver operating characteristic (ROC) curve of OS time was conducted for assessing the predictive performance of this gene signature *via* survival ROC package. The area under the curve (AUC) value of ROC was calculated to obtain the optimal model. Also, the Akaike information criterion (AIC) value of each point on the AUC was determined to distinguish the optimal cut-off point to differentiate the high- or low-risk patients.

### Assessment of Chemotherapy Drug Sensitivity

By employing the Genomics of Drug Sensitivity in Cancer (GDSC) database (www.cancerRxgene.org) (Yang et al., 2013), underlying chemotherapy drugs that were sensitive for osteosarcoma subjects harboring risk scores were predicted utilizing the pRRophetic package in the TARGET database (Geeleher et al., 2014).

### Connectivity Map

The cMap database contains over 7,000 expression profiling of human cells treated with small molecules (Lamb et al., 2006). DEGs between high- and low-risk groups were screened with the screening criteria of |FC| > 1.5 and adjusted *p* < 0.05. These up- and down-regulated genes were mapped onto the cMap database. The connectivity scores ranging from −1 to 1 indicated the correlations between small molecules and DEGs. The positive connectivity scores were indicative of stimulative effects of compounds on these DEGs and the negative connectivity scores were indicative of repressed effects of compounds on them. The candidate small molecules related to osteosarcoma were screened with |connectivity score|>0.9 and *p* < 0.05. Furthermore, shared mechanism of action (MOA) was predicted for these candidate drugs.

### Single-Sample Gene Set Enrichment Analysis

The infiltrations and activations of immune cells that were retrieved from published gene signature across all cancer as well as normal specimens were quantified utilizing the ssGSEA algorithm (Charoentong et al., 2017; Jia et al., 2018). The immune cells contained activated B cells, activated CD4 T cells, activated CD8 T cells, central memory CD4 T cells, central memory CD8 T cells, effector memory CD4 T cells, effector memory CD8 T cells, gamma delta T cells, immature B cells, memory B cells, regulatory T cells, T follicular helper cells, type 1 T helper cells, type 17 T helper cells, type 2 T helper cells, activated dendritic cells, CD56bright natural killer cells, CD56dim natural killer cells, eosinophils, immature dendritic cells, macrophages, mast cells, MDSCs, monocytes, natural killer cells, natural killer T cells, neutrophils, and plasmacytoid dendritic cells. The ssGSEA scores of each immune cell type were normalized in all samples in the TARGET dataset. Previous two studies have characterized marker genes of three phenotypes of osteosarcoma cell lines: tumorigenic phenotype, invasive phenotype, and colony forming phenotype (Lauvrak et al., 2013; Sharma et al., 2017). This study curated the shared marker genes in above studies, which played important roles in modulating the three phenotypes of osteosarcoma cell lines. The ssGSEA score of each phenotype was quantified in the TARGET dataset.

### Gene Set Enrichment Analysis

The KEGG pathways that were distinctly related to the risk score were analyzed by GSEA software (https://www.broadinstitute.org/gsea/index.jsp) based on the gene expression (Subramanian et al., 2005). The “c2.cp.kegg.v7.1.symbols.gmt” file was obtained as a reference gene set. Enrichment adjusted *p*-values were based on 1,000 permutations. Terms with |normalized enrichment score (NES)|>1 and adjusted *p* < 0.05 were recognized as significant enrichment.

### Nomogram

To better apply this prognostic gene signature, a nomogram was established in the training set for prediction of 1-, 3-, and 5-years survival probability of osteosarcoma patients.

### Unsupervised Clustering Analysis by Nonnegative Matrix Factorization

Consensus clustering analysis was performed with the NMF algorithm for identifying distinct molecular subtypes according to the 272 stromal–immune score-based DEGs. The expressions of these signatures (matrix A) were factorized into three nonnegative matrices. Repeated factorization of matrix A was carried out, and its outputs were aggregated for obtaining consensus clustering of osteosarcoma specimens in the TARGET dataset. The optimal number of clustering was chosen based on cophenetic, dispersion, as well as silhouette coefficients. The 200 nruns was utilized for performing the consensus clustering.

### Statistical Analysis

All analysis was presented using R version 3.4.1 (http://www.R-project.org) and its appropriate packages. Two groups were compared utilizing Wilcoxon test, while multiple comparisons were evaluated by applying Kruskal–Wallis test. Two-sided *p* < 0.05 indicated statistical significance. The source code of this study is provided in Supplementary Table S3.

## Results

### Stromal and Immune Scores Are Associated With Survival Outcomes of Osteosarcoma Patients

**FIGURE 2:**
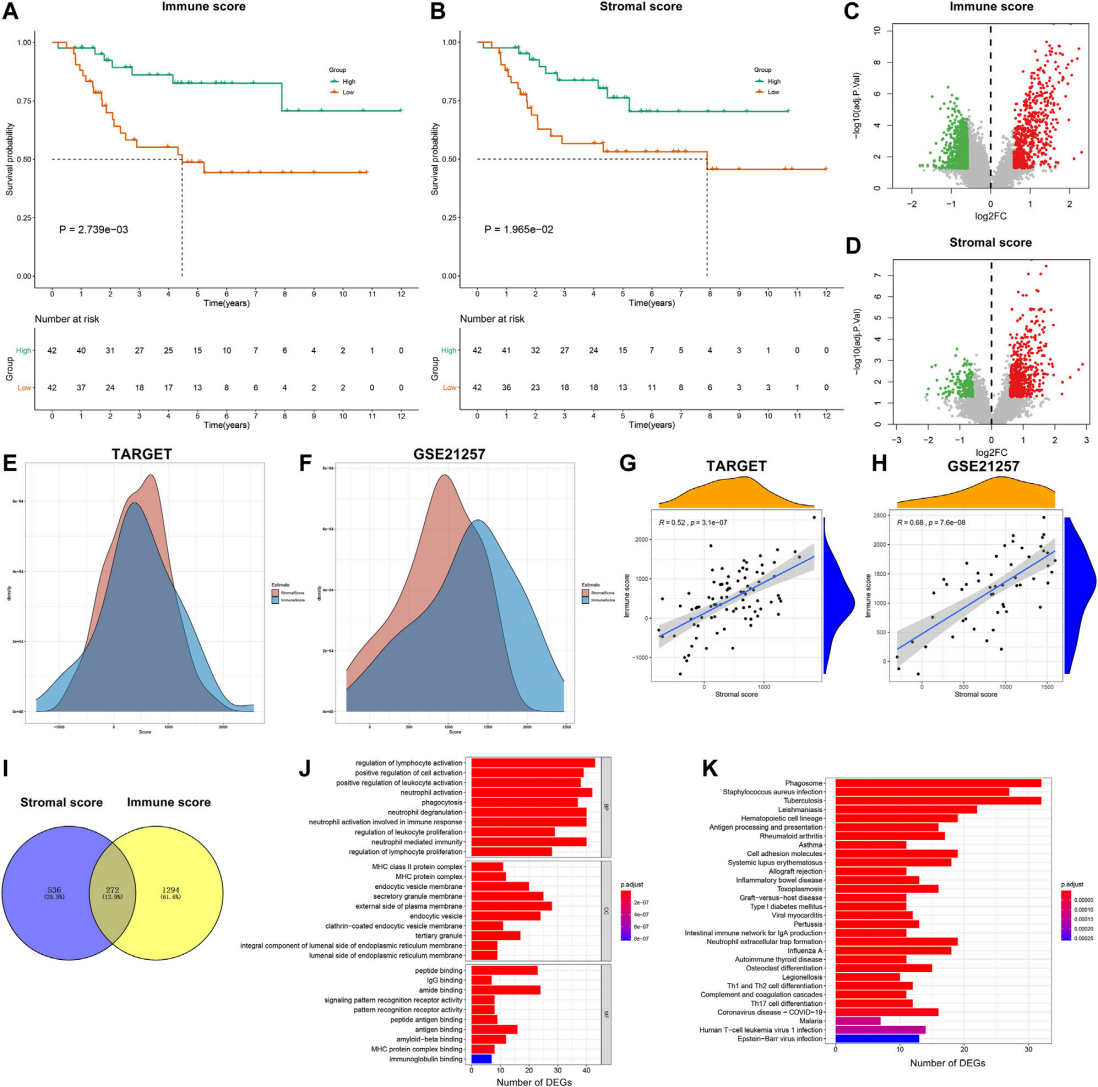
Stromal and immune scores are associated with survival outcomes of osteosarcoma patients. **(A,B)** Kaplan–Meier curves of overall survival (OS) between high and low **(A)** immune and **(B)** stromal score groups in the Therapeutically Applicable Research to Generate Effective Treatments (TARGET) database. The statistical differences were compared by log-rank test. The dash lines mean the corresponding survival time when survival probability was 0.5. **(C,D)** Volcano plots of up- (red) and downregulated (green) genes between high and low **(C)** immune and **(D)** stromal score groups in the TARGET database. **(E, F)** The distributions of stromal score and immune score across osteosarcoma samples in the TARGET and GSE21257 datasets. **(G,H)** Correlations between stromal score and immune score across osteosarcoma samples in the TARGET and GSE21257 datasets. **(I)** Venn diagram for stromal–immune score-based differentially expressed genes (DEGs) in osteosarcoma. **(J)** Gene ontology (GO) including biological process (BP), cellular component (CC) and molecular function (MF) and **(K)** Kyoto Encyclopedia of Genes and Genomes (KEGG) enrichment analysis results of stromal-immune score-based DEGs.

From the TARGET database, stromal and immune scores of each osteosarcoma sample were estimated by the ESTIMATE algorithm. All patients were classified into high and low stromal/immune score groups on the basis of the median values. The survival differences between groups were compared by Kaplan–Meier curves. Accordingly, patients with high immune scores (*p* = 2.739e-03) or stromal scores (*p* = 1.965e-02) displayed the advantages of survival duration (Figures 2A,B). To obtain stromal- or immune score-related genes, differential expression analyses were carried out between high and low stromal- or immune-score groups. As a result, 633 genes were upregulated and 933 genes were downregulated between high and low immune score groups (Figure 2C; Supplementary Table S4). Furthermore, there were 576 upregulated genes and 232 downregulated genes between high and low stromal score groups (Figure 2D; Supplementary Table S5). Also, we depicted the distributions of stromal score and immune score across osteosarcoma samples in the TARGET and GSE21257 datasets (Figures 2E,F). The correlation analyses showed that there were positive interactions between stromal score and immune score across osteosarcoma samples in the TARGET (R = 0.52 and *p* = 3.1e-07) and GSE21257 (R = 0.68 and *p* = 7.6e-08) datasets (Figures 2G,H). Following intersection, we obtained 272 stromal-immune score-based DEGs that showed consistent trends between high/low immune score groups and high/low stromal score groups in osteosarcoma (Figure 2I). Their biological functions were analyzed in depth. Accordingly, they were involved in regulating immune cell activation like lymphocytes and neutrophils (Figure 2J). MHC protein complex, antigen binding, and immunoglobulin were significantly enriched by these genes. Also, immune pathways such as antigen processing and presentation, Th1, Th2, and Th17 cell differentiation were distinctly enriched by these genes (Figure 2K).

### Development and Validation of a Stromal–Immune Score-based Prognostic Gene Signature for Osteosarcoma

**FIGURE 3:**
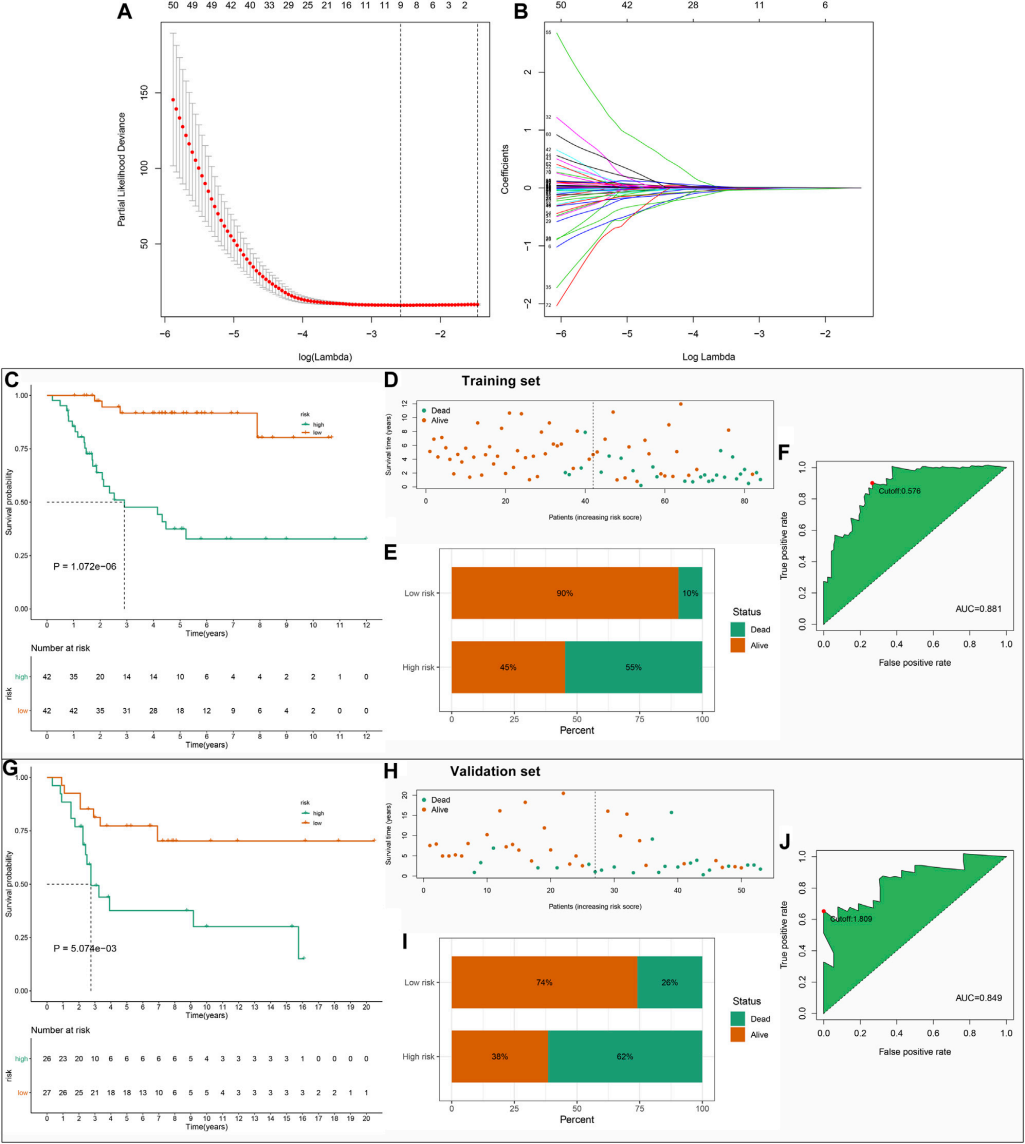
Establishment and verification of a stromal–immune score-based prognostic gene signature for osteosarcoma. **(A)** A 10-fold cross-verification for determining the number of factors. **(B)** Least absolute shrinkage and selection operator (LASSO) coefficient profiles of stromal–immune score-based gene features. **(C)** Kaplan–Meier curves of OS between high- and low-risk groups in the TARGET dataset. **(D,E)** The distribution and proportion of survival status in the high- and low-risk groups. The dotted line indicates the median value of risk scores. **(F)** Receiver operating characteristic (ROC) of OS time based on the risk scores in the TARGET dataset. The maximum inflection point was the cut-off point by the Akaike information criterion (AIC). **(G)** Kaplan–Meier curves of OS between high- and low-risk groups in the GSE21257 dataset. **(H,I)** The distribution and proportion of survival status of high- and low-risk osteosarcoma patients. **(J)** ROC of OS time for validating the predictive efficacy of this signature in the GSE21257 dataset.

By applying univariate Cox regression analysis, 85 genes were distinctly related to osteosarcoma prognosis among the 272 stromal- and immune score-related DEGs in the TARGET dataset (Table 1). Based on them, nine factors were included in this LASSO model in the training set, containing FPR1, GBP1, FUCA1, PDK1, BNIP3, EVI2B, APBB1IP, FOLR2, and COCH (Figures 3A,B). The risk score of each subject was determined according to the expression of FPR1 * (−0.00618173524670456) + expression of GBP1 * (−0.00428251209641429) + expression of FUCA1 * (−0.00768782441651179) + expression of PDK1 * 0.0078776021549243 + expression of BNIP3 * 0.00311883814864424 + expression of EVI2B * (−0.0014871562280857) + expression ofAPBB1IP * (−0.0258746909192034) + expression of FOLR2 * (−0.000566207076062648) + expression of COCH * 0.00731886943085428. All patients in the TARGET cohort were classified into high- and low-risk groups. In Figure 3C, patients in the high-risk group displayed the worse survival outcomes in comparison with those in the low-risk group (= 1.072e-06). Furthermore, there was higher proportion of dead patients in the high-risk group (Figures 3D,E). To assess the predictive efficacy of this signature, ROC of OS time was conducted. The AUC value was 0.881, demonstrating the well performance on predicting osteosarcoma patients’ prognosis (Figure 3F). The maximum inflection point was recognized as the cut-off point on the ROC curve by the AIC values. The GSE21257 dataset was used for independently validating this prognostic signature. Consistently, high-risk score indicated poor prognosis of osteosarcoma patients (*p* = 5.074e-03; Figure 3G). Higher proportion of dead patients was found in the high-risk group (Figures 3H,I). The AUC of OS was 0.849, confirming that this signature possessed high accuracy and sensitivity on predicting prognosis (Figure 3J).

**TABLE 1:** Univariate Cox regression analysis for prognosis-related stromal-immune score-based genes in osteosarcoma. The bold value represent the signature-related genes.

ID	HR	HR.95L	HR.95H	p	ID	HR	HR.95L	HR.95H	p
DOK3	0.885	0.816	0.961	0.004	LAIR1	0.945	0.898	0.993	0.027
CYP2S1	0.724	0.529	0.991	0.044	PRKCH	0.925	0.86	0.995	0.036
ESPN	1.07	1.019	1.124	0.006	GPR65	0.688	0.487	0.972	0.034
FAAH	1.028	1.002	1.055	0.037	CASP1	0.939	0.887	0.993	0.027
CPM	0.901	0.823	0.985	0.022	ARHGAP25	0.842	0.753	0.942	0.003
**FPR1**	0.819	0.702	0.956	0.011	ARHGAP30	0.9	0.82	0.988	0.027
ENG	0.994	0.988	0.999	0.033	CD163	0.956	0.922	0.991	0.015
FTL	0.9998	0.9996	0.99999	0.04	CD209	0.915	0.843	0.992	0.032
NPL	0.934	0.877	0.994	0.033	MS4A4A	0.946	0.908	0.987	0.01
**GBP1**	0.971	0.949	0.993	0.012	SLC7A7	0.941	0.899	0.985	0.009
GJA5	0.843	0.747	0.951	0.006	CEBPA	0.902	0.831	0.979	0.013
GJA4	0.961	0.923	0.9999	0.049	GIMAP1	0.814	0.668	0.993	0.043
GYPC	0.979	0.961	0.997	0.026	ACSL5	0.858	0.738	0.998	0.046
**FUCA1**	0.962	0.936	0.989	0.006	**EVI2B**	0.949	0.914	0.985	0.005
VSIG4	0.965	0.937	0.994	0.018	SMAD9	1.033	1.009	1.059	0.007
TYROBP	0.998	0.996	0.999	0.009	HCLS1	0.976	0.956	0.997	0.025
DOCK2	0.87	0.764	0.992	0.038	CSF3R	0.789	0.625	0.996	0.046
FCER1G	0.996	0.993	0.999	0.011	LGALS9	0.973	0.949	0.998	0.036
LCP2	0.923	0.857	0.994	0.035	IL10RA	0.91	0.834	0.992	0.033
TNFSF8	0.693	0.524	0.918	0.011	LILRB2	0.884	0.797	0.98	0.019
LY96	0.968	0.938	0.998	0.038	LILRB5	0.773	0.613	0.976	0.03
LY86	0.933	0.872	0.998	0.043	**APBB1IP**	0.927	0.884	0.971	0.002
MFNG	0.893	0.799	0.998	0.047	HAVCR2	0.951	0.906	0.9997	0.049
MPP1	0.954	0.926	0.984	0.003	APOL2	0.977	0.96	0.994	0.009
MSR1	0.969	0.939	0.999	0.045	LRRC25	0.921	0.851	0.995	0.037
NCF4	0.939	0.887	0.994	0.031	ARHGDIB	0.994	0.989	0.999	0.023
FCGR2A	0.985	0.971	0.999	0.03	ARHGAP9	0.901	0.824	0.985	0.022
FCGR2B	0.775	0.607	0.99	0.041	SAMHD1	0.979	0.963	0.995	0.012
POU2F2	0.887	0.794	0.991	0.034	RASGRP4	0.618	0.435	0.88	0.008
**PDK1**	1.078	1.033	1.124	6E-04	SLC43A2	0.88	0.791	0.979	0.019
PLEK	0.944	0.9	0.99	0.018	APOE	0.999	0.998	0.99997	0.042
CSF2RB	0.804	0.647	0.9998	0.05	BIN2	0.851	0.733	0.988	0.034
**BNIP3**	1.008	1.004	1.012	2E-04	C1QB	0.997	0.994	0.9997	0.032
TLR2	0.882	0.781	0.996	0.042	C1QA	0.997	0.995	0.99987	0.039
IL2RA	0.552	0.331	0.922	0.023	C1QC	0.998	0.996	0.9998	0.029
VAV1	0.871	0.776	0.977	0.018	CD14	0.994	0.988	0.99999	0.05
C3AR1	0.946	0.903	0.99	0.016	CD37	0.917	0.849	0.991	0.028
PILRA	0.932	0.871	0.996	0.038	CD53	0.984	0.97	0.997	0.018
SLCO2B1	0.957	0.922	0.993	0.02	**FOLR2**	0.985	0.976	0.995	0.004
PPFIA4	1.108	1.034	1.186	0.003	**COCH**	1.017	1.007	1.026	5E-04
PIK3R5	0.794	0.657	0.959	0.017	CTSS	0.98	0.961	0.9997	0.047
ITGAM	0.864	0.771	0.967	0.011	CYBB	0.975	0.952	0.9997	0.047
NFAM1	0.84	0.712	0.991	0.038					

### Prediction of Chemotherapy Drug Sensitivity and Potential Small Molecule Compounds Based on Risk Scores

**FIGURE 4:**
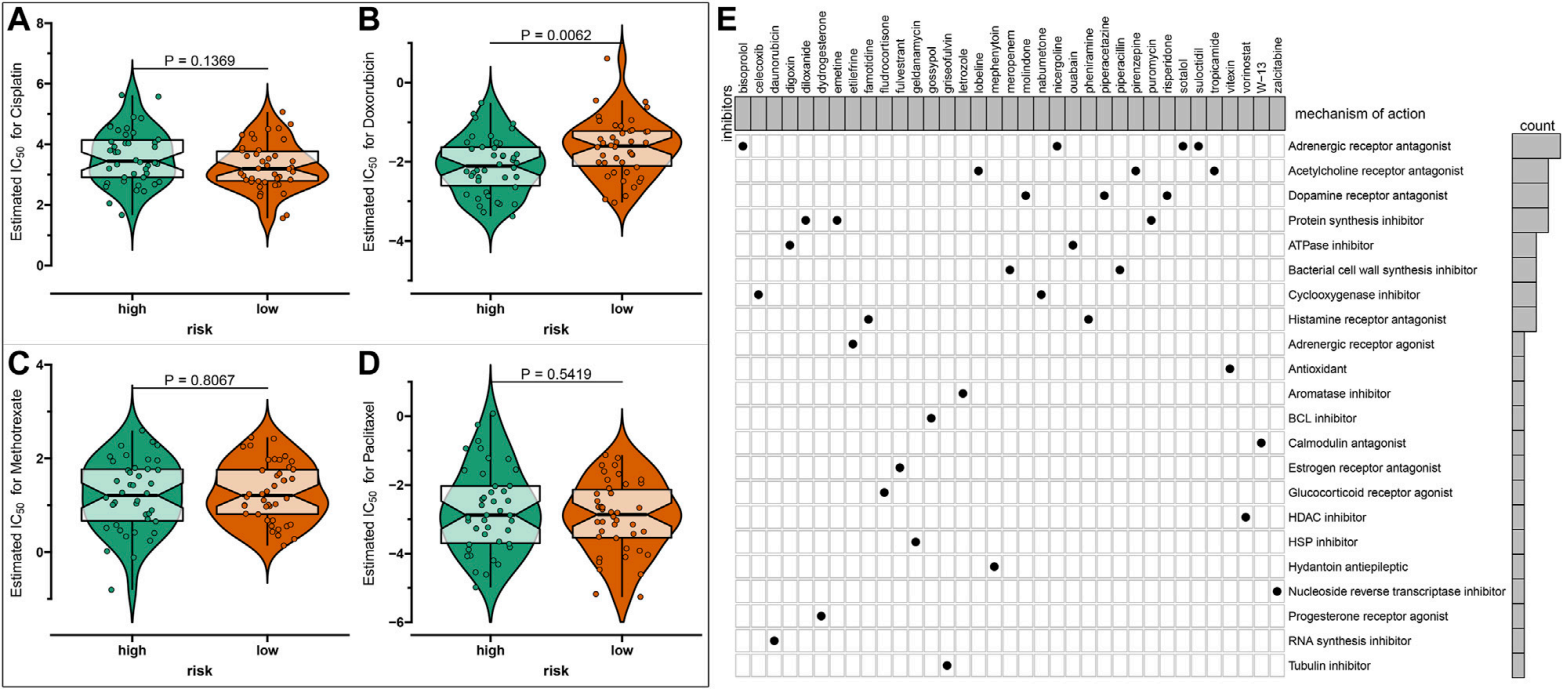
Prediction of chemotherapy drug sensitivity and potential small molecule compounds based on risk scores in osteosarcoma. **(A–D)** Drug sensitivity of **(A)** paclitaxel, **(B)** methotrexate, **(C)** doxorubicin, and **(D)** cisplatin between high- and low-risk osteosarcoma samples by the Genomics of Drug Sensitivity in Cancer (GDSC) database. **(E)** The shared mechanism of action among small molecule compounds.

The sensitivity to chemotherapy drugs including paclitaxel, methotrexate, doxorubicin, and cisplatin was estimated in each osteosarcoma sample from the TARGET database (Figures 4A–D). Accordingly, there were significantly lowered IC50 values of doxorubicin in the high-risk group than the low-risk group (*p* = 0.0062), indicating that the high-risk osteosarcoma patients were more sensitive to doxorubicin. There were 57 upregulated and 658 downregulated genes in the high-risk specimens than the low-risk specimens (Supplementary Table S6). Based on these DEGs, 61 small molecule compounds with |connectivity score| > 0.9 and *p* < 0.05 were predicted for treating osteosarcoma by cMap analysis (Table 2). Also, we analyzed the shared MOA among these small molecule compounds. As a result, sotalol, bisoprolol, suloctidil, and nicergoline shared adrenergic receptor antagonist (Figure 4E). Pirenzepine, tropicamide and lobeline had the shared acetylcholine receptor antagonist. Risperidone, molindone, and piperacetazine shared dopamine receptor antagonist. Protein synthesis inhibitor was shared by puromycin, emetine, and diloxanide.

**TABLE 2:** Potential small molecule drugs for osteosarcoma by connectivity map (cMap) analysis.

CMap name	Cell line	Mean	N	Enrichment	p	Specificity	Percent non-null
Zalcitabine	MCF7	0.728	2	0.979	0.00076	0.0057	100
Monensin	PC3	0.679	2	0.974	0.00109	0.0056	100
Tolnaftate	MCF7	0.707	2	0.971	0.00139	0.0115	100
Cefapirin	MCF7	0.698	2	0.97	0.00159	0	100
Sulfamonomethoxine	MCF7	0.677	2	0.97	0.00165	0.0065	100
Podophyllotoxin	MCF7	0.674	2	0.963	0.00235	0.0209	100
Amantadine	MCF7	0.68	2	0.962	0.00256	0	100
Gossypol	PC3	0.699	2	0.96	0.00266	0.023	100
Stachydrine	MCF7	0.721	2	0.957	0.00326	0	100
Pheniramine	MCF7	0.674	2	0.955	0.00374	0.0062	100
16,16-Dimethylprostaglandin E2	PC3	0.629	2	0.953	0.004	0.0229	100
Furazolidone	MCF7	0.646	2	0.951	0.00439	0	100
Metronidazole	MCF7	0.673	2	0.949	0.00483	0.022	100
Pivmecillinam	MCF7	0.632	2	0.948	0.00487	0.0119	100
Zardaverine	MCF7	0.688	2	0.946	0.00545	0.0272	100
Gabexate	MCF7	0.685	2	0.945	0.00567	0.0065	100
Metampicillin	PC3	0.612	2	0.944	0.00587	0.0162	100
Levopropoxyphene	MCF7	0.63	2	0.939	0.00714	0.0116	100
Etilefrine	MCF7	0.615	2	0.935	0.00797	0.0064	100
Brinzolamide	MCF7	0.734	2	0.935	0.00809	0.0063	100
Pyrithyldione	MCF7	0.622	2	0.932	0.00873	0.0294	100
Moroxydine	MCF7	0.754	2	0.931	0.00915	0.0296	100
Bretylium tosilate	MCF7	0.617	2	0.929	0.00968	0	100
Melatonin	MCF7	0.628	2	0.928	0.00984	0.0298	100
Iohexol	MCF7	0.576	2	0.927	0.01022	0.025	100
Khellin	MCF7	0.657	2	0.92	0.01276	0.0275	100
Pentoxifylline	PC3	0.564	2	0.914	0.01443	0.0165	100
Cloxacillin	MCF7	0.607	2	0.914	0.01465	0.012	100
Indoprofen	MCF7	0.569	2	0.911	0.01589	0.0345	100
Cefaclor	MCF7	0.618	2	0.909	0.01682	0.0396	100
Hydroflumethiazide	PC3	0.59	2	0.909	0.01696	0.0317	100
Oxolamine	MCF7	0.585	2	0.908	0.01752	0.0189	100
Benzathine Benzylpenicillin	MCF7	0.606	2	0.904	0.01881	0.0455	100
Rotenone	PC3	0.56	2	0.9	0.0204	0.0292	100
Adipiodone	MCF7	−0.733	2	−0.97	0.00191	0	100
Guaifenesin	PC3	−0.728	2	−0.959	0.00364	0.005	100
Octopamine	MCF7	−0.693	2	−0.953	0.00481	0.0188	100
Clorgiline	MCF7	−0.745	2	−0.952	0.00497	0.0074	100
Meropenem	MCF7	−0.731	2	−0.951	0.00525	0	100
Protoveratrine A	MCF7	−0.714	2	−0.95	0.00541	0.0161	100
Trolox C	MCF7	−0.711	2	−0.95	0.00551	0	100
Salsolidin	MCF7	−0.682	2	−0.944	0.00692	0.0177	100
Pipenzolate bromide	MCF7	−0.671	2	−0.94	0.00754	0.0245	100
Nicergoline	MCF7	−0.722	2	−0.937	0.00817	0.0132	100
Androsterone	MCF7	−0.763	2	−0.937	0.00823	0.0058	100
Seneciphylline	MCF7	−0.672	2	−0.937	0.00829	0.0378	100
Trazodone	MCF7	−0.752	2	−0.935	0.00891	0.0279	100
Sulfametoxydiazine	MCF7	−0.662	2	−0.928	0.0108	0	100
Dexpanthenol	MCF7	−0.684	2	−0.928	0.0109	0.0063	100
Procainamide	MCF7	−0.672	2	−0.927	0.01099	0.0154	100
Vitexin	MCF7	−0.676	2	−0.924	0.01183	0.0101	100
Pridinol	MCF7	−0.707	2	−0.922	0.01217	0.0058	100
Salbutamol	MCF7	−0.683	2	−0.918	0.01368	0.0064	100
Halcinonide	MCF7	−0.645	2	−0.918	0.01386	0.0084	100
Morantel	MCF7	−0.701	2	−0.917	0.01398	0.0049	100
Ritodrine	MCF7	-−0.68	2	−0.917	0.01416	0.0054	100
Corbadrine	MCF7	−0.718	2	−0.915	0.01467	0.0114	100
Betaxolol	MCF7	−0.669	2	−0.909	0.0167	0.0055	100
Aminophenazone	MCF7	−0.671	2	−0.906	0.01767	0.0057	100
Glycocholic acid	MCF7	−0.736	2	−0.905	0.01821	0.0124	100
Oxetacaine	MCF7	−0.624	2	−0.901	0.01944	0.0458	100

### Associations Between Risk Scores and Tumor Immune Microenvironment of Osteosarcoma

**FIGURE 5:**
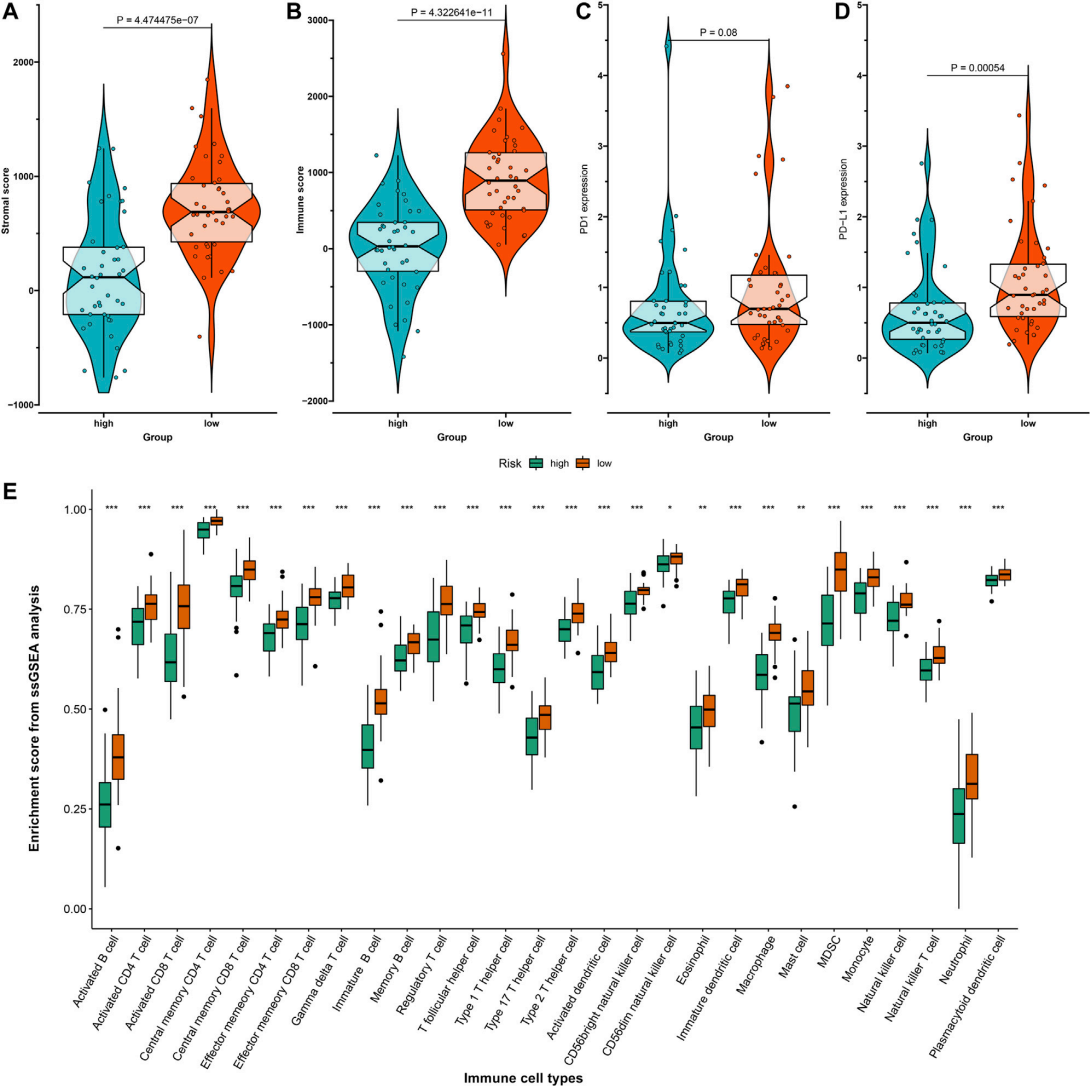
Associations between risk scores and tumor immune microenvironment of osteosarcoma in the TARGET dataset. **(A,B)** The distribution of **(A)** stromal scores and **(B)** immune scores in high- and low-risk osteosarcoma samples with the Estimation of Stromal and Immune Cells in Malignant Tumor Tissues Using Expression Data (ESTIMATE) algorithm. **(C,D)** The distribution of **(C)** programmed death-1 (PD-1) and **(D)** programmed death-ligand 1 (PD-L1) expression in high- and low-risk osteosarcoma samples. **(E)** The relative infiltration levels of immune cells in high- and low-risk groups with the single-sample gene set enrichment analysis (ssGSEA) algorithm. *p < 0.05; **p < 0.01; ***p < 0.001.

Stromal and immune scores were evaluated in the high- and low-risk osteosarcoma specimens with the ESTIMATE algorithm. Accordingly, higher stromal scores (*p* = 4.47e-07) and immune scores (*p* = 4.32e-11) were detected in the low-risk group compared to the high-risk group (Figures 5A,B). Considering that programmed death-1 (PD-1) and programmed death-ligand 1 (PD-L1) are well-established markers for prediction of the responses to anti-PD-1/L1 therapies (Sun et al., 2018), the expression distributions of PD-1 and PD-L1 were assessed. No significant difference in PD-1 expression was found between the high- and low-risk groups (Figure 5C). The low-risk osteosarcoma group displayed distinctly higher PD-L1 expression than the high-risk group (*p* = 0.00054; Figure 5D), indicating that the low-risk samples could be likely to respond to anti-PD-L1 therapy. The low-risk samples were enriched by various immune cells including activated B cells, activated CD4 T cells, activated CD8 T cells, central memory CD4 T cells, central memory CD8 T cells, effector memory CD4 T cells, effector memory CD8 T cells, gamma delta T cells, immature B cells, memory B cells, regulatory T cells, T follicular helper cells, type 1 T helper cells, type 17 T helper cells, type 2 T helper cells, activated dendritic cells, CD56bright natural killer cells, CD56dim natural killer cells, eosinophils, immature dendritic cells, macrophages, mast cells, MDSCs, monocytes, natural killer cells, natural killer T cells, neutrophils, and plasmacytoid dendritic cells (Figure 5E).

### Activation of Signaling Pathways Associated With the Gene Signature

**FIGURE 6:**
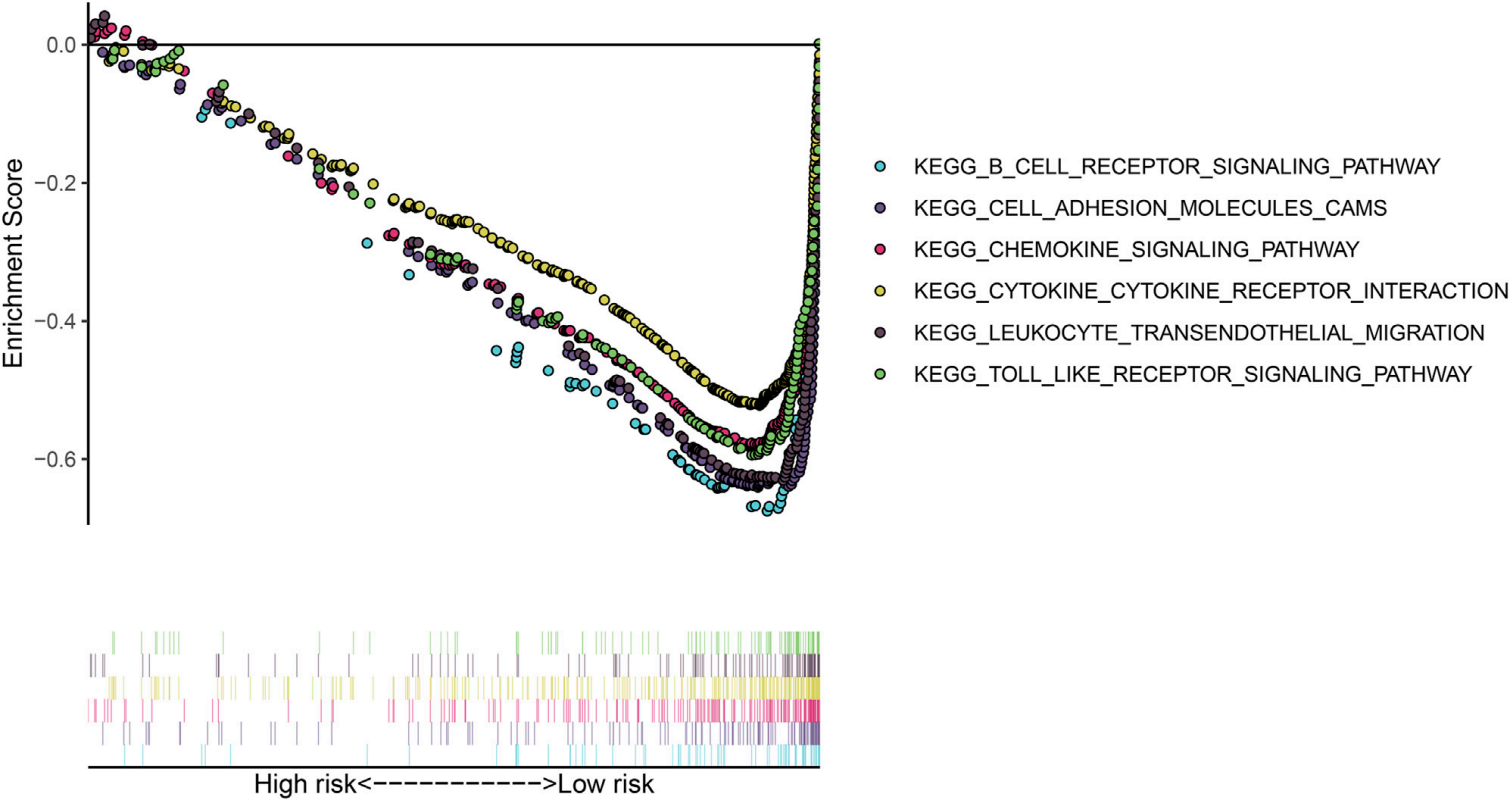
Gene set enrichment analysis (GSEA) for the KEGG pathways associated with the gene signature.

GSEA was utilized for exploring KEGG pathways associated with the gene signature. Under the threshold of |NES|>1 and adjusted *p* < 0.05, no pathways were significantly enriched in the high-risk osteosarcoma samples. B cell receptor signaling pathway (NES = −2.17, *p* < 0.0001), cell adhesion molecules cams (NES = −2.22, *p* = 0.001), chemokine signaling pathway (NES = −2.11, *p* = 0.003), cytokine-cytokine receptor interaction (NES = −2.16, *p* = 0.002), leukocyte transendothelial migration (NES = −2.30, *p* < 0.0001) and Toll-like receptor signaling pathway (NES = −2.10, *p* = 0.004) were significantly activated in the low-risk group (Figure 6).

### Associations Between Risk Scores and Clinicopathological Characteristics and Phenotypes of Osteosarcoma Cell Lines

**FIGURE 7:**
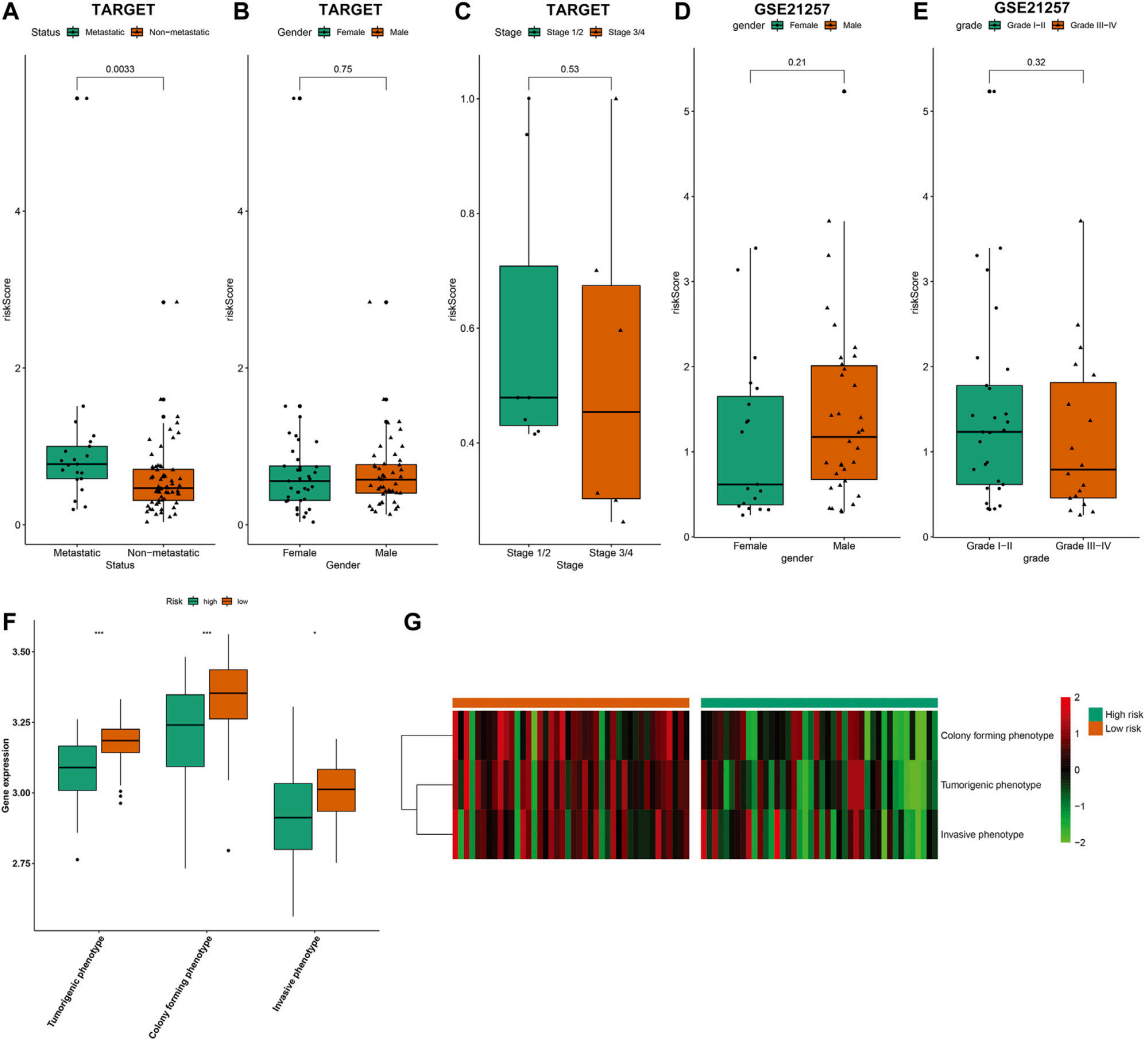
Associations between risk scores and clinicopathological characteristics and phenotypes of osteosarcoma cell lines. **(A–C)** Comparisons of risk score **(A)** between metastatic and non-metastatic patients; **(B)** between female and male patients; **(C)** between stage 1/2 and stage 3/4 patients in the TARGET cohort. **(D,E)** Comparisons of risk score **(D)** between female and male patients; **(E)** between grades I–II and grades III–IV patients in the GSE21257 dataset. **(F,G)** Comparisons of three phenotypes of osteosarcoma cell lines (tumorigenic phenotype, invasive phenotype, and colony forming phenotype) between high- and low-risk groups using ssGSEA method in the TARGET cohort. *p < 0.05; ***p < 0.001.

In TARGET cohort, we evaluated the associations between risk scores and clinicopathological characteristics of osteosarcoma patients. In Figure 7A, metastatic patients exhibited distinctly increased risk score than non-metastatic patients (*p* = 0.0033). However, no significant difference in risk score was found between female and male (Figure 7B) as well as between stage 1/2 and stage 3/4 (Figure 7C). In the GSE21257 dataset, we observed that there was no significant difference in risk score between female and male (Figure 7D) as well as between grades I–II and grades III–IV (Figure 7E). Three phenotypes of osteosarcoma cell lines were scored in each osteosarcoma sample in the TARGET cohort based on the relevant marker genes using ssGSEA method. We observed that risk score was negatively correlated to tumorigenic phenotype, invasive phenotype, and colony forming phenotype across osteosarcoma (Figures 7F,G).

### Establishment of a Nomogram for Prediction of 1-, 3-, and 5-years Survival of Osteosarcoma

**FIGURE 8:**
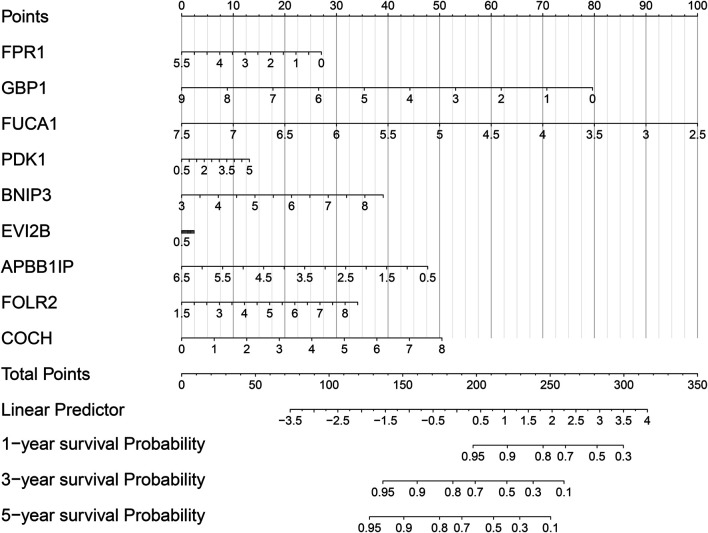
Establishment of a nomogram for prediction of 1-, 3-, and 5-years survival of osteosarcoma in the TARGET cohort.

For better applying this risk signature, a nomogram was established for osteosarcoma prognosis in the TARGET cohort, which contained FPR1, GBP1, FUCA1, PDK1, BNIP3, EVI2B, APBB1IP, FOLR2, and COCH. Our data demonstrated that this nomogram could be predictive of 1-, 3-, and 5-years survival probability of osteosarcoma patients (Figure 8). In this model, the 1-, 3-, and 5-years survival probability of the patients was determined through the total points that were calculated through adding up the point of each gene.

### Characterization of Osteosarcoma Subtypes With Distinct Prognostic Implications

**FIGURE 9:**
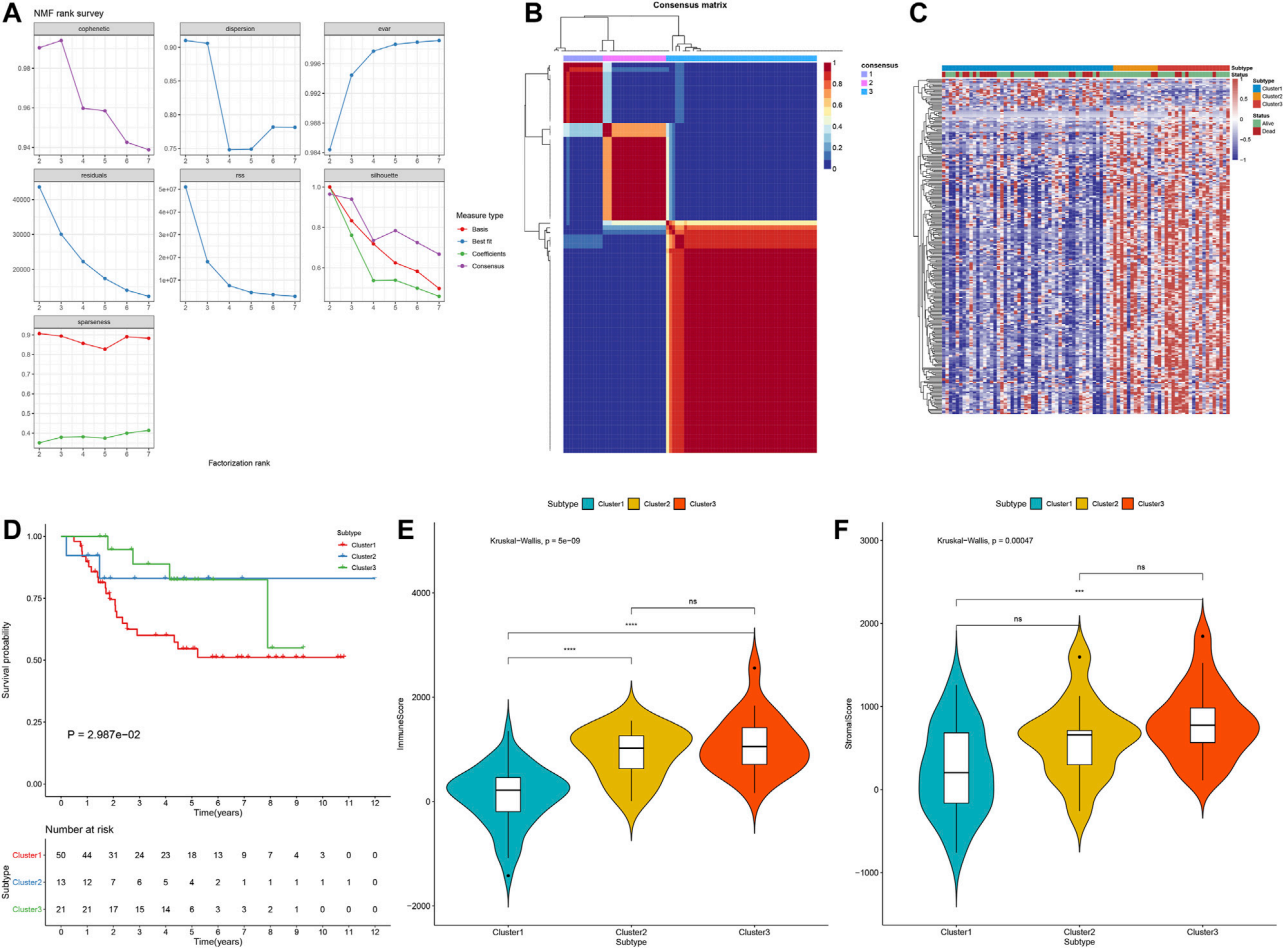
Establishment of osteosarcoma subtypes with distinct prognostic implications in the TARGET cohort based on the 272 stromal-immune score-based DEGs with the NMF algorithm. **(A)** The relationships between cophenetic, dispersion, evar, residuals, rss, silhouette and sparseness coefficients, and the number of clustering. **(B)** Heat map of nonnegative matrix factorization (NMF) clustering results of stromal-immune score-based signatures with clustering number = 3. **(C)** The heatmap of the differences in expression of the 272 stromal-immune score-based DEGs among three clusters. **(D)** Kaplan–Meier curves of OS for osteosarcoma patients in the TARGET cohort with three molecular subtypes. P for log-rank test. **(E)** The immune scores in three stromal–immune score-based clusters using the ESTIMATE algorithm. **(F)** The stromal scores of three gene clusters with the ESTIMATE algorithm. The differences between three subtypes were compared by Kruskal–Wallis test. ns, no statistical significance. ***p < 0.001; ****p < 0.0001.

Using the 272 stromal-immune score-based DEGs, consensus clustering analysis was applied with the NMF algorithm for stratifying osteosarcoma samples into distinct molecular subtypes. Accordingly, three molecular subtypes were characterized, including cluster1 (*n* = 50), cluster2 (*n* = 13) and cluster3 (*n* = 21; Figures 9A,B). To understand the difference in the underlying biology of the three subtypes, we established a heatmap that showed the differences in expression of the 272 DEGs among three clusters (Figure 9C). Among them, cluster1 had the worst survival outcomes (*p* = 2.987e-02; Figure 9D). Also, we noticed distinct differences in immune and stromal scores between molecular patterns. The lowest immune scores (p = 5e-09; Figure 9E) and stromal scores (*p* = 0.00047; Figure 9F) were found in cluster1.

### Stromal–Immune Score-based Modification Patterns Characterize Distinct Immune Landscapes

**FIGURE 10:**
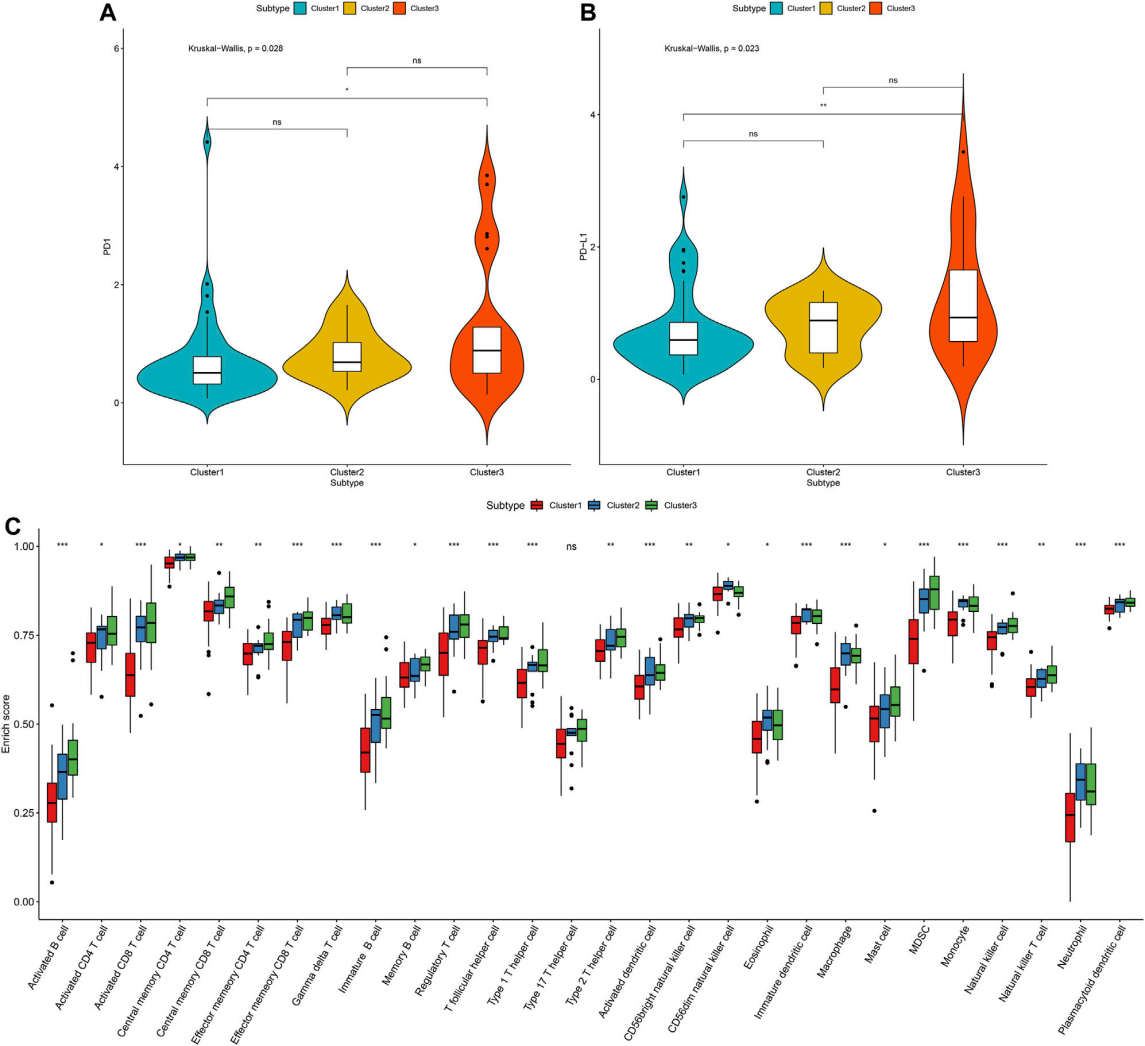
Tumor immune microenvironment characteristics in distinct stromal–immune score-based modification patterns for osteosarcoma in the TARGET cohort. **(A)** Comparisons of PD-1 expression across three stromal-immune score-based gene clusters. **(B)** Relative distribution of PD-L1 expression in three clusters. The differences between three subtypes were compared by Kruskal–Wallis test. **(C)** The fractions of tumor-infiltrating immune cells in three clusters with the ssGSEA algorithm. The statistical differences between clusters were compared with the Kruskal–Wallis test. ns, no statistical significance. *p < 0.05; **p < 0.01; ***p < 0.001.

This study further investigated the distributions of PD1 and PD-L1 expression across three stromal-immune score-based gene clusters. Among them, cluster3 had the highest PD-1 expression (*p* = 0.028; Figure 10A). Moreover, this cluster exhibited the highest PD-L1 expression (*p* = 0.023; Figure 10B). In Figure 10C, the three molecular patterns were characterized by distinct immune cell infiltrations. Cluster3 exhibited the highest infiltration levels of activated B cells (*p* < 0.001), activated CD4 T cells (*p* < 0.05), activated CD8 T cells (*p* < 0.001), central memory CD4 T cells (*p* < 0.05), central memory CD8 T cells (*p* < 0.01), effector memory CD4 T cells (*p* < 0.01), effector memory CD8 T cells (*p* < 0.001), gamma delta T cells (*p* < 0.001), immature B cells (*p* < 0.001), memory B cells (*p* < 0.05), regulatory T cells (*p* < 0.001), T follicular helper cells (*p* < 0.001), type 1 T helper cells (*p* < 0.001), type 2 T helper cells (*p* < 0.01), activated dendritic cells (*p* < 0.001), mast cells (*p* < 0.05), MDSC (*p* < 0.001), natural killer cells (*p* < 0.001), natural killer T cells (*p* < 0.01) and plasmacytoid dendritic cells (*p* < 0.001), while cluster1 had the lowest infiltration levels of above immune cells. Thus, cluster3 was recognized as a “hot” tumor, cluster2 was recognized as an “excluded” tumor, and cluster1 was recognized as a “cold” tumor.

## Discussion

Osteosarcoma, a common bone malignancy, is prone to metastasis as well as undesirable survival outcomes (Yao et al., 2018). Stromal and immune cells in the tumor microenvironment are critical regulators of osteosarcoma progression, drug resistance and treatment response (Smeland et al., 2019). Based on immune–stromal score-based DEGs, we developed and verified a gene signature for the prediction of survival outcomes of osteosarcoma patients as well as characterized three distinct molecular subtypes.

An immune-stromal score-based gene signature containing FPR1, GBP1, FUCA1, PDK1, BNIP3, EVI2B, APBB1IP, FOLR2, and COCH was established for predicting clinical outcomes of osteosarcoma subjects. Consistent with previous research, FUCA1 was distinctly correlated to osteosarcoma patients (Yiqi et al., 2020). PDK1 upregulation was detected in osteosarcoma, which elevated a proliferative capacity of osteosarcoma cells (Li et al., 2017). EVI2B that possessed the predictive potential on prognosis was associated with metastasis and immune cell infiltration in osteosarcoma (Yang et al., 2021). Immune-related APBB1IP was in relation to clinical outcomes of osteosarcoma patients (Cao et al., 2020). More assays should be carried out to verify their biological implications in osteosarcoma. Consistently, high stromal or immune scores were in relation to prolonged survival time of osteosarcoma (Alves et al., 2019). Patients with high risk were indicative of unfavorable prognosis. After external verification, this signature possessed the well performance on prediction of clinical outcomes of osteosarcoma patients.

At current, neoadjuvant chemotherapy and surgery are approved to remove primary or metastatic osteosarcoma, followed by adjuvant chemotherapy after surgery, which may increase OS time of osteosarcoma patients (Zhang et al., 2020). Nevertheless, drug resistance leads to worse clinical outcomes (Xiao et al., 2018). Doxorubicin represents the most effective first-line drug regarding high-grade osteosarcoma (Buondonno et al., 2019). Here, high-risk patients were more sensitive to doxorubicin, which indicated that these subjects were more likely to benefit from this chemotherapy drug. Immunotherapy, especially immune checkpoint inhibitors, has hugely altered the therapeutic landscape of metastatic or recurrent osteosarcoma (Chen et al., 2021). However, only some patients can benefit from immunotherapy (Yu et al., 2019). Here, we found that low-risk patients had the increase in PD-L1 expression and higher infiltration levels of various immune cells, indicating that these patients were more likely to respond to anti-PD-L1 therapy. Our GSEA results also confirmed that immune pathways such as B cell receptor signaling pathway (Long et al., 2016), chemokine signaling pathway (Chao et al., 2020), cytokine-cytokine receptor interaction (Lu et al., 2018), and toll-like receptor signaling pathway (Zhou et al., 2020a) were significantly activated in the low-risk patients.

Previous two studies have characterized the gene set of phenotypes of 22 osteosarcoma cell lines, including tumorigenic phenotype, invasive phenotype, and colony forming phenotype, which reflect the osteosarcoma heterogeneity (Lauvrak et al., 2013; Sharma et al., 2017). One has characterized osteosarcoma phenotypes at multiple levels. The other suggests that these phenotypically differentiated osteosarcoma cell lines play important roles in modulating tumor microenvironment by multitype network-guided target controllability analysis (Sharma et al., 2017). Here, we employed ssGSEA method to quantify tumorigenic phenotype, invasive phenotype, and colony-forming phenotype across osteosarcoma samples based on the expression profiles of shared marker genes between above two studies. We observed that immune-stromal score-based risk score was negatively associated with tumorigenic phenotype, invasive phenotype, and colony-forming phenotype, indicative of the important role of the risk score in the progression of osteosarcoma.

By employing NMF algorithm, we established three immune-stromal score-based gene patterns for osteosarcoma patients, with distinct survival outcomes. Among them, cluster3 had the highest PD-1/PD-L1 expression and the highest infiltration levels of activated B cells, activated CD4 T cells, activated CD8 T cells, central memory CD4 T cells, central memory CD8 T cells, effector memory CD4 T cells, effector memory CD8 T cells, gamma delta T cells, immature B cells, memory B cells, regulatory T cells, T follicular helper cells, type 1 T helper cells, type 2 T helper cells, activated dendritic cells, mast cells, MDSC, natural killer cells, natural killer T cells and plasmacytoid dendritic cells. These data indicated that osteosarcoma patients in cluster3 were more likely to benefit from immunotherapy. The limitations of this study should be pointed out. First, due to limited number of patients, their clinical implications will be validated in a larger osteosarcoma cohort in our future studies. Second, in the TARGET and GSE21257 datasets, there was lack of clinicopathological characteristics (such as stage and grade) of osteosarcoma patients. Thus, it was difficult to analyze the correlations between the risk score and clinicopathological characteristics.

## Conclusion

Collectively, this study established an immune–stromal score-based gene signature and distinct molecular subtypes, which could predict the survival outcomes of osteosarcoma patients sensitive to chemotherapy and immunotherapy. Thus, the risk score may assist decision making for individualized therapy and follow-up project in clinical practice.

## Data Availability

The original contributions presented in the study are included in the article/Supplementary Material, further inquiries can be directed to the corresponding authors.

## Supplementary Material

Supplementary Table 1 The specific clinical information of 84 samples in the TARGET database.

Supplementary Table 2 The specific clinical information of 53 samples in the GSE21257 dataset.

Supplementary Table 3 The source code of this study.

Supplementary Table 4 Immune score-based differentially expressed genes in osteosarcoma.

Supplementary Table 5 Stromal score-based differentially expressed genes in osteosarcoma.

Supplementary Table 6 Differentially expressed genes between high and low risk osteosarcoma groups.

## Abbreviations

AUC, area under the curve; BP, biological process; CC, cellular component; CMap, Connectivity Map; DEGs, differentially expressed genes; ESTIMATE, Estimation of Stromal and Immune cells in Malignant Tumor tissues using Expression data; FC, fold change; GDSC, Genomics of Drug Sensitivity in Cancer; GEO, Gene Expression Omnibus; GO, Gene Ontology; GSEA, Gene set enrichment analysis; KEGG, Kyoto Encyclopedia of Genes and Genomes; LASSO, least absolute shrinkage and selection operator; MF, molecular function; NES, normalized enrichment score; NMF, nonnegative matrix factorization; OS, overall survival; PD-1, programmed death-1; PD-L1, programmed death-ligand 1. ROC, receiver operating characteristic; ssGSEA. Single-sample gene set enrichment analysis; TARGET, Therapeutically Applicable Research to Generate Effective Treatments.
