# Supplementary material for: Analysis of Immune–Stromal Score-Based Gene Signature and Molecular Subtypes in Osteosarcoma: Implications for Prognosis and Tumor Immune Microenvironment
Source: Front Genet. 2021 Sep 23;12:699385. doi: 10.3389/fgene.2021.699385 (PMC8495166; doi:10.3389/fgene.2021.699385)
Supplement: Supplementary file 4 [file Table3.DOCX]

library(limma)

library(estimate)

filterCommonGenes(input.f="uniq.symbol.txt",

output.f="commonGenes.gct",

id="GeneSymbol")

estimateScore(input.ds = "commonGenes.gct",

output.ds="estimateScore.gct")

scores=read.table("estimateScore.gct",skip = 2,header = T)

rownames(scores)=scores[,1]

scores=t(scores[,3:ncol(scores)])

rownames(scores)=gsub("\\.","\\-",rownames(scores))

out=rbind(ID=colnames(scores),scores)

write.table(out,file="scores.txt",sep="\t",quote=F,col.names=F)

#survival analysis

library(survival)

library(survminer)

data<-read.table("ImmuneScore.txt",head=T,sep='\t',check.names = F,row.names = 1);data$OS=data$OS/365

diff=survdiff(Surv(OS, Status) ~Group,data = data)

pValue=1-pchisq(diff$chisq,df=1)

pValue=signif(pValue,4)

pValue=format(pValue, scientific = TRUE)

fit <- survfit(Surv(OS, Status) ~Group, data = data)

ggsurvplot(fit,

data=data,

conf.int=F,

pval=paste0 ("P = ",pValue),

pval.size=6,

legend.title="Group",

legend.labs=levels(factor(data[,"Group"])),

legend = c(0.8, 0.8),

font.legend=12,

xlab="Time(years)",

break.time.by = 1,

palette = "Dark2",

surv.median.line = "hv",

risk.table=T,

cumevents=F,

risk.table.height=.25)

library(survival)

library(survminer)

data<-read.table("StromalScore.txt",head=T,sep='\t',check.names = F,row.names = 1);data$OS=data$OS/365

diff=survdiff(Surv(OS, Status) ~Group,data = data)

pValue=1-pchisq(diff$chisq,df=1)

pValue=signif(pValue,4)

pValue=format(pValue, scientific = TRUE)

fit <- survfit(Surv(OS, Status) ~Group, data = data)

ggsurvplot(fit,

data=data,

conf.int=F,

pval=paste0 ("P = ",pValue),

pval.size=6,

legend.title="Group",

legend.labs=levels(factor(data[,"Group"])),

legend = c(0.8, 0.8),

font.legend=12,

xlab="Time(years)",

break.time.by = 1,

palette = "Dark2",

surv.median.line = "hv",

risk.table=T,

cumevents=F,

risk.table.height=.25)

#Diff analysis

logFoldChange=0.58

adjustP=0.05

conNum=42

treatNum=42

library(limma)

#Immunescore

rt<-read.table("uniq.symbol.txt",head=T,sep='\t',check.names = F,row.names = 1)

rt=log2(rt+1)

df<-read.table("ImmuneScore.txt",head=T,sep='\t',check.names = F,row.names = 1)

sample<-intersect(row.names(df),colnames(rt))

rt<-rt[,sample]

modType=c(rep("con",conNum),rep("treat",treatNum))

design <- model.matrix(~0+factor(modType))

colnames(design) <- c("con","treat")

fit <- lmFit(rt,design)

cont.matrix<-makeContrasts(treat-con,levels=design)

fit2 <- contrasts.fit(fit, cont.matrix)

fit2 <- eBayes(fit2)

allDiff=topTable(fit2,adjust='fdr',number=200000)

write.table(allDiff,file="mrnaAll.xls",sep="\t",quote=F)

#write table

Immunescore_diffSig <- allDiff[with(allDiff, (abs(logFC)>logFoldChange & adj.P.Val < adjustP )), ]

diffSigOut=rbind(id=colnames(Immunescore_diffSig),Immunescore_diffSig)

write.table(diffSigOut,file="mrnaDiff.xls",sep="\t",quote=F,col.names=F)

write.table(diffSigOut,file="mrnaDiff.txt",sep="\t",quote=F,col.names=F)

pdf(file="mrnaVol.pdf",height=5,width=5)

xMax=max(abs(allDiff$logFC))

yMax=max(-log10(allDiff$adj.P.Val))

plot(allDiff$logFC, -log10(allDiff$adj.P.Val), xlab="logFC",ylab="-log10(adj.P.Val)",

main="Volcano", ylim=c(0,yMax),xlim=c(-xMax,xMax),yaxs="i",pch=20, cex=0.8)

diffSub=subset(allDiff, adj.P.Val<adjustP & logFC>logFoldChange)

points(diffSub$logFC, -log10(diffSub$adj.P.Val), pch=20, col="red",cex=0.8)

diffSub=subset(allDiff, adj.P.Val<adjustP & logFC<(-logFoldChange))

points(diffSub$logFC, -log10(diffSub$adj.P.Val), pch=20, col="green",cex=0.8)

abline(v=0,lty=2,lwd=3)

dev.off()

#Stromalscore

rt<-read.table("uniq.symbol.txt",head=T,sep='\t',check.names = F,row.names = 1)

rt=log2(rt+1)

df<-read.table("StromalScore.txt",head=T,sep='\t',check.names = F,row.names = 1)

sample<-intersect(row.names(df),colnames(rt))

rt<-rt[,sample]

modType=c(rep("con",conNum),rep("treat",treatNum))

design <- model.matrix(~0+factor(modType))

colnames(design) <- c("con","treat")

fit <- lmFit(rt,design)

cont.matrix<-makeContrasts(treat-con,levels=design)

fit2 <- contrasts.fit(fit, cont.matrix)

fit2 <- eBayes(fit2)

allDiff=topTable(fit2,adjust='fdr',number=200000)

write.table(allDiff,file="mrnaAll.xls",sep="\t",quote=F)

#write table

StromalScore_diffSig <- allDiff[with(allDiff, (abs(logFC)>logFoldChange & adj.P.Val < adjustP )), ]

diffSigOut=rbind(id=colnames(StromalScore_diffSig),StromalScore_diffSig)

write.table(diffSigOut,file="mrnaDiff.xls",sep="\t",quote=F,col.names=F)

write.table(diffSigOut,file="mrnaDiff.txt",sep="\t",quote=F,col.names=F)

pdf(file="mrnaVol.pdf",height=5,width=5)

xMax=max(abs(allDiff$logFC))

yMax=max(-log10(allDiff$adj.P.Val))

plot(allDiff$logFC, -log10(allDiff$adj.P.Val), xlab="logFC",ylab="-log10(adj.P.Val)",

main="Volcano", ylim=c(0,yMax),xlim=c(-xMax,xMax),yaxs="i",pch=20, cex=0.8)

diffSub=subset(allDiff, adj.P.Val<adjustP & logFC>logFoldChange)

points(diffSub$logFC, -log10(diffSub$adj.P.Val), pch=20, col="red",cex=0.8)

diffSub=subset(allDiff, adj.P.Val<adjustP & logFC<(-logFoldChange))

points(diffSub$logFC, -log10(diffSub$adj.P.Val), pch=20, col="green",cex=0.8)

abline(v=0,lty=2,lwd=3)

dev.off()

#intersect gene

intersect_gene<-intersect(row.names(Immunescore_diffSig),row.names(StromalScore_diffSig))

library("clusterProfiler")

library("org.Hs.eg.db")

library("enrichplot")

library("ggplot2")

#GO analysis

kk <- enrichGO(gene = intersect_gene,

OrgDb = org.Hs.eg.db,

pvalueCutoff =0.05,

qvalueCutoff = 0.05,

ont="all",

keyType = 'ENSEMBL')

write.table(kk,file="GO.txt",sep="\t",quote=F,row.names = F)

pdf(file="barplot.pdf",width = 10,height = 8)

barplot(kk, drop = TRUE, showCategory =10,split="ONTOLOGY") + facet_grid(ONTOLOGY~., scale='free')

dev.off()

#KEGG analysis

gene.df <- bitr(intersect_gene, fromType = "SYMBOL",

toType = c"ENTREZID",

OrgDb = org.Hs.eg.db)

KEGG <- enrichKEGG(gene = gene.df$V1,

organism = 'hsa',

pvalueCutoff = 0.05)

pdf(file="barplot.pdf",width = 10,height = 8)

barplot(KEGG, showCategory =30)

dev.off()

write.table(KEGG,file="KEGG.txt",sep="\t",quote=F,row.names = F)

library(survival)

pFilter=0.05

rt=read.table("expTime.txt",header=T,sep="\t",check.names=F,row.names=1)

outTab=data.frame()

sigGenes=c("OS","Status")

for(i in colnames(rt[,3:ncol(rt)])){

cox <- coxph(Surv(OS, Status) ~ rt[,i], data = rt)

coxSummary = summary(cox)

coxP=coxSummary$coefficients[,"Pr(>|z|)"]

if(coxP<pFilter){

sigGenes=c(sigGenes,i)

outTab=rbind(outTab,

cbind(id=i,

HR=coxSummary$conf.int[,"exp(coef)"],

HR.95L=coxSummary$conf.int[,"lower .95"],

HR.95H=coxSummary$conf.int[,"upper .95"],

pvalue=coxSummary$coefficients[,"Pr(>|z|)"])

)

}

}

write.table(outTab,file="uniCox.txt",sep="\t",row.names=F,quote=F)

uniSigExp=rt[,sigGenes]

uniSigExp=cbind(id=row.names(uniSigExp),uniSigExp)

write.table(uniSigExp,file="uniSigExp.txt",sep="\t",row.names=F,quote=F)

library("glmnet")

library("survival")

rt=read.table("uniSigExp.txt",header=T,sep="\t",row.names=1,check.names=F)

rt$OS=rt$OS/365

x=as.matrix(rt[,c(3:ncol(rt))])

y=data.matrix(Surv(rt$OS,rt$Status))

fit <- glmnet(x, y, family = "cox")

pdf("lambda.pdf")

plot(fit, xvar = "lambda", label = TRUE)

dev.off()

cvfit <- cv.glmnet(x, y, family="cox", nfolds=10)

pdf("cvfit.pdf")

plot(cvfit)

abline(v=log(c(cvfit$lambda.min,cvfit$lambda.1se)),lty="dashed")

dev.off()

coef <- coef(fit, s = cvfit$lambda.min)

index <- which(coef != 0)

actCoef <- coef[index]

lassoGene=row.names(coef)[index]

geneCoef=cbind(Gene=lassoGene,Coef=actCoef)

write.table(geneCoef,file="geneCoef.txt",sep="\t",quote=F,row.names=F)

riskScore=predict(cvfit, newx = x, s = "lambda.min",type="response")

outCol=c("OS","Status",lassoGene)

risk=as.vector(ifelse(riskScore>median(riskScore),"high","low"))

outTab=cbind(rt[,outCol],riskScore=as.vector(riskScore),risk)

write.table(cbind(id=rownames(outTab),outTab),

file="trainRisk.txt",

sep="\t",

quote=F,

row.names=F)

z=read.table("test.txt",header=T,sep="\t",row.names=1,check.names=F)

z=z[,c("OS","Status",as.vector(gene[,1]))]

s=as.matrix(z[,c(3:ncol(z))])

riskScore=predict(cvfit, newx = s, s = "lambda.min",type="response")

outCol=c("OS","Status",lassoGene)

risk=as.vector(ifelse(riskScore>median(riskScore),"high","low"))

outTab=cbind(z[,outCol],riskScore=as.vector(riskScore),risk)

write.table(cbind(id=rownames(outTab),outTab),

file="testRisk.txt",

sep="\t",

quote=F,

row.names=F)

data<-read.table("trainRisk.txt",head=T,sep='\t',check.names = F,row.names = 1);data$OS=data$OS/365

diff=survdiff(Surv(OS, Status) ~risk,data = data)

pValue=1-pchisq(diff$chisq,df=1)

pValue=signif(pValue,4)

pValue=format(pValue, scientific = TRUE)

fit <- survfit(Surv(OS, Status) ~risk, data = data)

ggsurvplot(fit,

data=data,

conf.int=F,

pval=paste0 ("P = ",pValue),

pval.size=6,

legend.title="risk",

legend.labs=levels(factor(data[,"risk"])),

legend = c(0.8, 0.8),

font.legend=12,

xlab="Time(years)",

break.time.by = 1,

palette = "Dark2",

surv.median.line = "hv",

risk.table=T,

cumevents=F,

risk.table.height=.25)

data<-read.table("testRisk.txt",head=T,sep='\t',check.names = F,row.names = 1);data$OS=data$OS/365

diff=survdiff(Surv(OS, Status) ~risk,data = data)

pValue=1-pchisq(diff$chisq,df=1)

pValue=signif(pValue,4)

pValue=format(pValue, scientific = TRUE)

fit <- survfit(Surv(OS, Status) ~risk, data = data)

ggsurvplot(fit,

data=data,

conf.int=F,

pval=paste0 ("P = ",pValue),

pval.size=6,

legend.title="risk",

legend.labs=levels(factor(data[,"risk"])),

legend = c(0.8, 0.8),

font.legend=12,

xlab="Time(years)",

break.time.by = 1,

palette = "Dark2",

surv.median.line = "hv",

risk.table=T,

cumevents=F,

risk.table.height=.25)

library(plyr)

library(ggplot2)

library(corrplot)

bioBar=function(riskFile=null, pdfFile=null, corFile=null){

rt=read.table(riskFile,header=T,sep="\t",check.names=F,row.names=1)

rt$Status[rt$Status==0]="Alive"

rt$Status[rt$Status==1]="Dead"

rt$risk[rt$risk=="low"]="Low risk"

rt$risk[rt$risk=="high"]="High risk"

rt1=rt[,c("Status","risk")]

df=as.data.frame(table(rt1))

df$Status = factor(df$Status, levels=c("Dead","Alive"))

df=ddply(df, .(risk), transform, percent = Freq/sum(Freq) * 100)

df=ddply(df, .(risk), transform, pos = (cumsum(Freq) - 0.5 * Freq))

df$label=paste0(sprintf("%.0f", df$percent), "%")

p=ggplot(df, aes(x = factor(risk), y = percent, fill = Status)) +

geom_bar(position = position_stack(), stat = "identity", width = .7) +

scale_fill_manual(values=c("red","green"))+

xlab("")+ ylab("Percent")+ guides(fill=guide_legend(title=" Status"))+

geom_text(aes(label = label), position = position_stack(vjust = 0.5), size = 3) +

coord_flip()+

theme_bw()

pdf(file=pdfFile, width=6, height=3)

print(p)

dev.off()

}

bioBar(riskFile="trainRisk.txt", pdfFile="train.barplot.pdf")

bioBar(riskFile="testRisk.txt", pdfFile="test.barplot.pdf")

library(survivalROC)

rt=read.table("trainRisk.txt", header=T, sep="\t", check.names=F, row.names=1)

predictTime=5

roc=survivalROC(Stime=rt$OS, status=rt$Status, marker=rt$riskScore, predict.time =predictTime, method="KM")

sum=roc$TP-roc$FP

cutOp=roc$cut.values[which.max(sum)]

cutTP=roc$TP[which.max(sum)]

cutFP=roc$FP[which.max(sum)]

pdf(file="Train_ROC.cutoff.pdf",width=5.5,height=5.5)

plot(roc$FP, roc$TP, type="l", xlim=c(0,1), ylim=c(0,1),col="black",

xlab="False positive rate", ylab="True positive rate",

lwd = 2, cex.main=1.2, cex.lab=1.2, cex.axis=1.2, font=1.2)

polygon(x=c(0,roc$FP,1,0),y=c(0,roc$TP,1,0),col="#24B35D",border=NA)

segments(0,0,1,1,lty=2)

points(cutFP,cutTP, pch=20, col="red",cex=1.5)

text(cutFP+0.15,cutTP-0.05,paste0("Cutoff:",sprintf("%0.3f",cutOp)))

text(0.85, 0.1, paste0("AUC=",sprintf("%.3f",roc$AUC)), cex=1.2)

dev.off()

library(survivalROC)

rt=read.table("testRisk.txt", header=T, sep="\t", check.names=F, row.names=1)

predictTime=5

roc=survivalROC(Stime=rt$OS, status=rt$Status, marker=rt$riskScore, predict.time =predictTime, method="KM")

sum=roc$TP-roc$FP

cutOp=roc$cut.values[which.max(sum)]

cutTP=roc$TP[which.max(sum)]

cutFP=roc$FP[which.max(sum)]

pdf(file="Test_ROC.cutoff.pdf",width=5.5,height=5.5)

plot(roc$FP, roc$TP, type="l", xlim=c(0,1), ylim=c(0,1),col="black",

xlab="False positive rate", ylab="True positive rate",

lwd = 2, cex.main=1.2, cex.lab=1.2, cex.axis=1.2, font=1.2)

polygon(x=c(0,roc$FP,1,0),y=c(0,roc$TP,1,0),col="#24B35D",border=NA)

segments(0,0,1,1,lty=2)

points(cutFP,cutTP, pch=20, col="red",cex=1.5)

text(cutFP+0.15,cutTP-0.05,paste0("Cutoff:",sprintf("%0.3f",cutOp)))

text(0.85, 0.1, paste0("AUC=",sprintf("%.3f",roc$AUC)), cex=1.2)

dev.off()

library(pheatmap)

rt=read.table("trainRisk.txt",sep="\t",header=T,row.names=1,check.names=F)

rt=rt[order(rt$riskScore),]

color=as.vector(rt$Status)

color[color==1]="#1B9E77"

color[color==0]="#D95F02"

pdf(file="trainRisk_survStat.pdf",width = 10,height = 4)

plot(rt$futime,

pch=19,

xlab="Patients (increasing risk socre)",

ylab="Survival time (years)",

col=color)

legend("topleft", c("Dead", "Alive"),bty="n",pch=19,col=c("#1B9E77","#D95F02"),cex=1.2)

abline(v=lowLength,lty=2)

dev.off()

library(pheatmap)

rt=read.table("testRisk.txt",sep="\t",header=T,row.names=1,check.names=F)

rt=rt[order(rt$riskScore),]

color=as.vector(rt$Status)

color[color==1]=""#1B9E77""

color[color==0]=""#D95F02""

pdf(file="trainRisk_survStat.pdf",width = 10,height = 4)

plot(rt$futime,

pch=19,

xlab="Patients (increasing risk socre)",

ylab="Survival time (years)",

col=color)

legend("topleft", c("Dead", "Alive"),bty="n",pch=19,col=c("#1B9E77","#D95F02"),cex=1.2)

abline(v=lowLength,lty=2)

dev.off()

logFoldChange=0.58

adjustP=0.05

conNum=42

treatNum=42

library(limma)

rt<-read.table("uniq.symbol.txt",head=T,sep='\t',check.names = F,row.names = 1)

rt=log2(rt+1)

df<-read.table("trainRisk.txt",head=T,sep='\t',check.names = F,row.names = 1)

sample<-intersect(row.names(df),colnames(rt))

rt<-rt[,sample]

modType=c(rep("con",conNum),rep("treat",treatNum))

design <- model.matrix(~0+factor(modType))

colnames(design) <- c("con","treat")

fit <- lmFit(rt,design)

cont.matrix<-makeContrasts(treat-con,levels=design)

fit2 <- contrasts.fit(fit, cont.matrix)

fit2 <- eBayes(fit2)

allDiff=topTable(fit2,adjust='fdr',number=200000)

write.table(allDiff,file="mrnaAll.xls",sep="\t",quote=F)

#write table

diffSig <- allDiff[with(allDiff, (abs(logFC)>logFoldChange & adj.P.Val < adjustP )), ]

diffSigOut=rbind(id=colnames(diffSig),diffSig)

write.table(diffSigOut,file="mrnaDiff.xls",sep="\t",quote=F,col.names=F)

write.table(diffSigOut,file="mrnaDiff.txt",sep="\t",quote=F,col.names=F)

#CMAP

#uploaded the down and up gene to the CMAP database and obtationed potential drugs clinical info

library(xlsx)

library(tidyverse)

library(GEOquery)

library(plyr)

library(circlize)

library(ComplexHeatmap)

options(java.parameters = "-Xmx8000m")

Sys.setenv(LANGUAGE = "en")

options(stringsAsFactors = FALSE)

MoAinput <- openxlsx::read.xlsx("Connectivity_Map_Output.xlsx", sheet = 1, colNames = T)

MoAinput[MoAinput$MoA == "NFkB pathway inhibitor", ]

PerturbagenID <- unlist(str_split(MoAinput$Name, ", "))

names(PerturbagenID) <- unlist(str_split(MoAinput$Perturbagen.Id, ", "))

MoAinput <- MoAinput[, c("MoA", "Perturbagen.Id")] %>% split(.$MoA) %>% lapply("[[", 2) %>%

lapply(., function(x)unlist(str_split(x, ", "))) %>% plyr::ldply(., data.frame)

colnames(MoAinput) <- c("mechanisms of action", "inhibitors")

oncoprintinput <- reshape2::dcast(MoAinput, `mechanisms of action` ~ inhibitors)

rownames(oncoprintinput) <- oncoprintinput$`mechanisms of action`

oncoprintinput <- oncoprintinput[, -1] %>% as.matrix(oncoprintinput)

oncoprintinput[!is.na(oncoprintinput)] <- "inhibitor"

oncoprintinput[is.na(oncoprintinput)] <- ""

colnames(oncoprintinput) <- PerturbagenID[colnames(oncoprintinput)]

oncoprintinput <- oncoprintinput[, order(colnames(oncoprintinput))]

alter_fun = list(

background = function(x, y, w, h)

grid.rect(x, y, w*0.9, h*0.9, gp = gpar(fill = "white", col = "grey")),

# dots

inhibitor = function(x, y, w, h)

grid.points(x, y, pch = 16, size = unit(0.8, "char"))

)

ha_coldata <- colSums(apply(oncoprintinput, 2, function(x) x=="inhibitor") + 0) %>% as.numeric()

ha_rowdata <- rowSums(apply(oncoprintinput, 2, function(x) x=="inhibitor") + 0) %>% as.numeric()

top_ha <- HeatmapAnnotation(inhibitors = anno_barplot(ha_coldata, axis = F, border = F,

gp = gpar(fill = "grey"),

bar_width = 1),

annotation_name_side = "left",

annotation_name_rot = 90)

right_ha <- rowAnnotation(count = anno_barplot(ha_rowdata, axis = F, border = F,

gp = gpar(fill = "grey"),

bar_width = 1, width = unit(1.5, "cm")),

annotation_name_side = "top",

annotation_name_rot = 0)

pdf("MoA.pdf", width = 10, height = 8, onefile = F)

oncoPrint(oncoprintinput, alter_fun = alter_fun,

show_column_names = TRUE, column_names_side = "top",

column_order = 1:ncol(oncoprintinput),

top_annotation = top_ha,

right_annotation = right_ha,

show_pct = FALSE, show_heatmap_legend = F)

decorate_annotation("inhibitors", {

grid.text("mechanism of action", unit(1, "npc") + unit(3, "mm"), just = "left")})

dev.off()

#Drug sensitivity

library(pRRophetic)

library(ggplot2)

library(cowplot)

dat <- read.table("uniq.symbol.txt",sep = "\t",row.names = 1,header = T,stringsAsFactors = F,check.names = F)

ann <- read.table("trainRisk.txt",sep = "\t",row.names = 1,header = T,stringsAsFactors = F,check.names = F)

GCP.drug <- read.table("drug.txt")

GCP.drug <- GCP.drug$V1

jco <- c("#EABF00", "#2874C5", "red")

GCPinfo <- GCP.IC50 <- GCP.expr <- cvOut <- predictedPtype <- predictedBoxdat <- list()

plotp <- list()

for (drug in GCP.drug) {

set.seed(1248103)

cat(drug," starts!\n")

predictedPtype[[drug]] <- pRRopheticPredict(testMatrix = as.matrix(dat[,rownames(ann)]),

drug = drug,

tissueType = "allSolidTumors",

selection = 1)

if(!all(names(predictedPtype[[drug]])==rownames(ann))) {stop("Name mismatched!\n")}

predictedBoxdat[[drug]] <- data.frame("est.ic50"=predictedPtype[[drug]],

"risk"=ann$risk ,

row.names = names(predictedPtype[[drug]]))

predictedBoxdat[[drug]]$risk <- factor(predictedBoxdat[[drug]]$risk,levels = c("high","low"),ordered = T)

p <- ggplot(data = predictedBoxdat[[drug]], aes(x=risk, y=est.ic50))

p <- p + geom_boxplot(aes(fill = risk)) +

scale_fill_manual(values = jco[1:length(unique(ann$risk))]) +

theme(legend.position="none") +

theme(axis.text.x = element_text(angle = 45, hjust = 1,size = 12),plot.title = element_text(size = 12, hjust = 0.5)) +

xlab("") + ylab("Estimated IC50") +

ggtitle(drug)

plotp[[drug]] <- p

cat(drug," has been finished!\n")

}

p2 <- plot_grid(plotlist=plotp, ncol=2)

ggsave("boxplot of predicted IC50_multiple.pdf", width = 8, height = 4)

p <- vector()

for (drug in GCP.drug) {

tmp <- wilcox.test(as.numeric(predictedBoxdat[[drug]][which(predictedBoxdat[[drug]]$risk %in% "high"),"est.ic50"]),

as.numeric(predictedBoxdat[[drug]][which(predictedBoxdat[[drug]]$risk %in% "low"),"est.ic50"]),alternative = "less")$p.value

p <- append(p,tmp)

}

names(p) <- GCP.drug

print(p)

gmtFile="immune.gmt"

library(GSVA)

library(limma)

library(GSEABase)

mat<-read.table("uniq.symbol.txt",head=T,sep='\t',check.names = F,row.names = 1)

mat<-as.matrix(mat)

geneSet=getGmt(gmtFile,

geneIdType=SymbolIdentifier())

ssgseaScore=gsva(mat, geneSet, method='ssgsea', kcdf='Gaussian', abs.ranking=TRUE)

normalize=function(x){

return((x-min(x))/(max(x)-min(x)))}

ssgseaOut=normalize(ssgseaScore)

ssgseaOut=rbind(id=colnames(ssgseaOut),ssgseaOut)

write.table(ssgseaOut,file="ssgseaOut.txt",sep="\t",quote=F,col.names=F)

library(reshape2)

library(ggpubr)

df<-read.table("ssgseaOut.txt",head=T,sep='\t',check.names=F,row.names=1)

data<-melt(df,

id.vars = c('Risk'),

measure.vars = colnames(df[-1]),

variable.name='Immune_cell',

value.name='Expression')

p=ggboxplot(data, x="Immune_cell", y="Expression", fill = "Risk",

ylab="Enrich score",

xlab="",

palette = "Dark2" )

p=p+rotate_x_text(60)

p+stat_compare_means(aes(group=Risk),symnum.args=list(cutpoints = c(0, 0.001, 0.01, 0.05, 1), symbols = c("***", "**", "*", "ns")),label = "p.signif")+theme(axis.text = element_text(size = 13, face = "bold"))

#Stromalscore/Immunescore

library(reshape2)

library(ggpubr)

my_comparisons<-list(c("high","low"))

df<-read.table("Estimate_comparison.txt",head=T,sep='\t',check.names = F,row.names = 1)

ggviolin(df, x = "Risk", y = "StromalScore", fill = "Risk",

palette = "Dark2",

add = "boxplot", add.params = list(fill = "white"))+

stat_compare_means(comparisons = my_comparisons)

ggviolin(df, x = "Risk", y = "ImmuneScore", fill = "Risk",

palette = "Dark2",

add = "boxplot", add.params = list(fill = "white"))+

stat_compare_means(comparisons = my_comparisons)

#Immune_checkpoint

df<-read.table("Immunecheckpoint_comparison.txt",head=T,sep='\t',check.names = F,row.names = 1)

ggviolin(df, x = "Risk", y = "PD1", fill = "Risk",

palette = "Dark2",

add = "boxplot", add.params = list(fill = "white"))+

stat_compare_means(comparisons = my_comparisons)

ggviolin(df, x = "Risk", y = "PD-L1", fill = "Risk",

palette = "Dark2",

add = "boxplot", add.params = list(fill = "white"))+

stat_compare_means(comparisons = my_comparisons)

# After run the GSEA software, we can obtained the enrichment files for each pathway

library(plyr)

library(ggplot2)

library(grid)

library(gridExtra)

files=grep(".xls",dir(),value=T)

data = lapply(files,read.delim)

names(data) = files

dataSet = ldply(data, data.frame)

dataSet$pathway = gsub(".xls","",dataSet$.id)

gseaCol=c("#58CDD9","#6E568C","#E0367A","#D8D155","#64495D","#7CC767","#223D6C","#D20A13","#FFD121","#088247","#11AA4D")

pGsea=ggplot(dataSet,aes(x=RANK.IN.GENE.LIST,y=RUNNING.ES,fill=pathway,group=pathway))+

geom_point(shape=21) + scale_fill_manual(values = gseaCol[1:nrow(dataSet)]) +

labs(x = "", y = "Enrichment Score", title = "") + scale_x_continuous(expand = c(0, 0)) +

scale_y_continuous(expand = c(0, 0),limits =c(min(dataSet$RUNNING.ES-0.02), max(dataSet$RUNNING.ES+0.02))) +

theme_bw() + theme(panel.grid =element_blank()) + theme(panel.border = element_blank()) +

theme(axis.line = element_line(colour = "black")) + theme(axis.line.x = element_blank(),axis.ticks.x = element_blank(),axis.text.x = element_blank()) +

geom_hline(yintercept = 0) + guides(fill=guide_legend(title = NULL)) +

theme(legend.background = element_blank()) + theme(legend.key = element_blank())

pGene=ggplot(dataSet,aes(RANK.IN.GENE.LIST,pathway,colour=pathway))+geom_tile()+

scale_color_manual(values = gseaCol[1:nrow(dataSet)]) +

labs(x = "High risk<----------->Low risk", y = "", title = "") +

scale_x_discrete(expand = c(0, 0)) + scale_y_discrete(expand = c(0, 0)) +

theme_bw() + theme(panel.grid = element_blank()) + theme(panel.border = element_blank()) + theme(axis.line = element_line(colour = "black"))+

theme(axis.line.y = element_blank(),axis.ticks.y = element_blank(),axis.text.y = element_blank())+ guides(color=FALSE)

gGsea = ggplot_gtable(ggplot_build(pGsea))

gGene = ggplot_gtable(ggplot_build(pGene))

maxWidth = grid::unit.pmax(gGsea$widths, gGene$widths)

gGsea$widths = as.list(maxWidth)

gGene$widths = as.list(maxWidth)

dev.off()

pdf('multipleGSEA.pdf',

width=9,

height=5)

par(mar=c(5,5,2,5))

grid.arrange(arrangeGrob(gGsea,gGene,nrow=2,heights=c(.8,.3)))

dev.off()

pbc<-read.table("Trainrisk.txt",head=T,sep='\t',check.names = F,row.names = 1)

pbc$died <- pbc$Status==1

head(pbc)

library(rms)

dd<-datadist(pbc)

options(datadist="dd")

options(na.action="na.delete")

summary(pbc$OS)

coxpbc<-cph(formula = Surv(OS,died) ~ FPR1 + GBP1 + FUCA1 + PDK1 + BNIP3 + EVI2B + APBB1IP + FOLR2 + COCH ,data=pbc,x=T,y=T,surv = T,na.action=na.delete) #,time.inc =2920

print(coxpbc)

surv<-Survival(coxpbc)

surv3<-function(x) surv(3,x)

surv1<-function(x) surv(1,x)

surv5<-function(x) surv(5,x)

x<-nomogram(coxpbc,fun = list(surv1,surv3,surv5),lp=T,

funlabel = c('1-year survival Probability','3-year survival Probability','5-year survival Probability'),

maxscale = 100,fun.at = c(0.95,0.9,0.8,0.7,0.6,0.5,0.4,0.3,0.2,0.1))

pdf("nomogram_classical.pdf",width = 12,height = 10)

plot(x, lplabel="Linear Predictor",

xfrac=.35,varname.label=TRUE, varname.label.sep="=", ia.space=.2,

tck=NA, tcl=-0.20, lmgp=0.3,

points.label='Points', total.points.label='Total Points',

total.sep.page=FALSE,

cap.labels=FALSE,cex.var = 1.6,cex.axis = 1.05,lwd=5,

label.every = 1,col.grid = gray(c(0.8, 0.95)))

dev.off()

library(stringr)

library(CancerSubtypes)

library(ConsensusClusterPlus)

library(limma)

data<-read.table("uniSigExp.txt",sep="\t",header=T,check.names=F,row.names=1)

data<-as.matrix(data)

results = nmf(data,2:7,nrun=30, .opt='v', seed=123456789)

plot(results)

results = nmf(data,3,nrun=30, .opt='v', seed=123456789)

W <- basis(results) ; H <- coef(results)

h1<-t(H)

colnames(h1)<-c("Cluster1","Cluster2","Cluster3") #三个类

j1 <- max.col(h1, "first")

value <- h1[cbind(1:nrow(h1), j1)]

cluster <- colnames(h1)[j1]

res <- data.frame(value, cluster)

row.names(res)<-row.names(h1)

write.table(res,"group.txt",quote=F,sep='\t')

data<-read.table("Cluster_survival.txt",head=T,sep='\t',check.names = F,row.names = 1)

diff=survdiff(Surv(OS, Status) ~Subtype,data = data)

pValue=1-pchisq(diff$chisq,df=1)

pValue=signif(pValue,4)

pValue=format(pValue, scientific = TRUE)

fit <- survfit(Surv(OS, Status) ~Subtype, data = data)

ggsurvplot(fit,

data=data,

conf.int=F,

pval=paste0 ("P = ",pValue),

pval.size=6,

legend.title="Subtype",

legend.labs=levels(factor(data[,"Subtype"])),

legend = c(0.8, 0.8),

font.legend=12,

xlab="Time(years)",

break.time.by = 1,

palette = "Dark2",

surv.median.line = "hv",

risk.table=T,

cumevents=F,

risk.table.height=.25)

df<-read.table("ssgseaOut.txt",head=T,sep='\t',check.names=F,row.names=1)

data<-melt(df,

id.vars = c('Subtype'),

measure.vars = colnames(df[-1]),

variable.name='Immune_cell',

value.name='Expression')

p=ggboxplot(data, x="Immune_cell", y="Expression", fill = "Subtype",

ylab="Enrich score",

xlab="",

palette = "Dark2" )

p=p+rotate_x_text(60)

p+stat_compare_means(aes(group=Subtype),symnum.args=list(cutpoints = c(0, 0.001, 0.01, 0.05, 1), symbols = c("***", "**", "*", "ns")),label = "p.signif")+theme(axis.text = element_text(size = 13, face = "bold"))

#Stromalscore/Immunescore

library(reshape2)

library(ggpubr)

my_comparisons<-list(c("Cluster1","Cluster2"),c("Cluster1","Cluster3"),c("Cluster2","Cluster3"))

df<-read.table("Estimate_comparison.txt",head=T,sep='\t',check.names = F,row.names = 1)

ggviolin(df, x = "Subtype", y = "StromalScore", fill = "Subtype",

palette = "Dark2",

add = "boxplot", add.params = list(fill = "white"))+

stat_compare_means(comparisons = my_comparisons)

ggviolin(df, x = "Subtype", y = "ImmuneScore", fill = "Subtype",

palette = "Dark2",

add = "boxplot", add.params = list(fill = "white"))+

stat_compare_means(comparisons = my_comparisons)

#Immune_checkpoint

df<-read.table("Immunecheckpoint_comparison.txt",head=T,sep='\t',check.names = F,row.names = 1)

ggviolin(df, x = "Subtype", y = "PD1", fill = "Subtype",

palette = "Dark2",

add = "boxplot", add.params = list(fill = "white"))+

stat_compare_means(comparisons = my_comparisons)

ggviolin(df, x = "Subtype", y = "PD-L1", fill = "Subtype",

palette = "Dark2",

add = "boxplot", add.params = list(fill = "white"))+

stat_compare_means(comparisons = my_comparisons)
